# Peroxidase‐Mimicking Nanozymes for Rapid Detection of Infectious Diseases

**DOI:** 10.1002/advs.202524259

**Published:** 2026-02-26

**Authors:** Shikuan Shao, Victor Fernandez‐Gonzalez, Xiaochuan Wang, Blake Pylipow, Claudia Herrera, Xiaohu Xia

**Affiliations:** ^1^ Department of Chemistry University of Central Florida Orlando Florida USA; ^2^ School of Social Work College of Health Professions and Sciences University of Central Florida Orlando Florida USA; ^3^ Department of Tropical Medicine and Infectious Disease School of Public Health and Tropical Medicine and Vector‐Borne and Infectious Disease Research Center Tulane University New Orleans Louisiana USA; ^4^ NanoScience Technology Center University of Central Florida Orlando Florida USA; ^5^ Biionix Cluster University of Central Florida Orlando Florida USA

**Keywords:** biosensing, catalysis, infectious disease, nanozyme, peroxidase

## Abstract

Infectious diseases pose a critical global health threat, and rapid and sensitive diagnostics are essential for effective intervention. Conventional methods are often slow, costly, and require centralized instrumentation and trained personnel, limiting timely decision‐making. Peroxidase‐mimicking nanozymes (PMNs) have emerged as powerful alternatives to natural peroxidases, enabling the development of rapid, sensitive, and robust diagnostic platforms. The superior performance of these platforms is rooted in the well‐defined structural and compositional features of PMNs, which endow them with high peroxidase‐like activity to catalyze substrate oxidation into easily detectable signals. Furthermore, the unique physicochemical properties of PMNs support the design of simplified workflows, miniaturized point‐of‐care devices, and novel sensing logics that are inaccessible to conventional protein enzymes. This review highlights structure–activity relationships guiding PMN design, synthetic strategies for constructing these nanozymes, their integration into biosensing platforms for infectious disease detection, and the role of artificial intelligence in PMN design and biosensing applications. By focusing on the interplay between PMN materials and sensing technologies, we illustrate how these systems advance rapid, sensitive, and cost‐effective diagnostics. We also provide our perspective on the broader societal impact of PMN‐enabled infectious disease diagnostics, along with the challenges and opportunities that shape their translation toward practical clinical use.

## Graphical abstract

Peroxidase‐mimicking nanozymes (PMNs) have emerged as robust and versatile materials for rapid infectious disease diagnostics. This review highlights the rational design and controlled synthesis of PMNs, summarizes key biomarkers relevant to infectious diseases, examines their integration into diverse rapid detection platforms, and highlights artificial intelligence in PMN design and diagnostics. We also discuss the cost‐effectiveness and potential societal impact of the PMN‐based diagnostic platforms, along with the challenges and opportunities for their clinical translation. Created with BioRender.com.

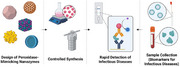

## 1 Introduction

Infectious diseases remain major threats to global health with both emerging and longstanding pathogens continuing to impose significant clinical and socioeconomic burdens [1, 2]. For example, *coronaviruses* such as SARS‐CoV‐2 continue to cause recurrent outbreaks worldwide, leading to respiratory illnesses that range from mild flu‐like symptoms to severe pneumonia and multi‐organ failure [3, 4]. *Human immunodeficiency virus* (HIV), as another example, represents a chronic infection that persists globally, where undiagnosed cases can silently progress to acquired immunodeficiency syndrome, resulting in severe immunosuppression and opportunistic infections [5]. Other infections, such as *Trypanosoma cruzi*, the causative agent of *Chagas* disease, pose additional challenges due to prolonged asymptomatic phases and frequent underdiagnosis, which increase the risk of chronic clinical complications [6, 7]. These examples show the critical importance of early diagnostics and screening, which enable timely intervention and help prevent uncontrolled transmission. Detecting SARS‐CoV‐2 at an early stage allows for patient isolation and targeted treatment to curb community spread [8], while identifying HIV infection during the asymptomatic phase permits the initiation of antiretroviral therapy (ART) [9, 10], improving long‐term outcomes and reducing transmission risk.

Currently, common diagnostic techniques for infectious diseases include nucleic acid‐based polymerase chain reaction (PCR), enzyme‐linked immunosorbent assay (ELISA), lateral flow assay (LFA), culture‐based methods, and histological or microscopic approaches [7, 11, 12, 13]. These methods have made tremendous contributions to disease prevention and clinical management, but they still face notable challenges. PCR offers high sensitivity and specificity, yet it requires prior sequence knowledge and cannot readily differentiate viable from non‐viable pathogens [11]. ELISA is widely adopted for large‐scale screening and relies on horseradish peroxidase (HRP) as a reporter enzyme that catalyzes the hydrogen peroxide (H_2_O_2_)‐mediated oxidation of chromogenic substrates to produce a colorimetric signal. However, HRP is a protein that is relatively expensive and prone to denaturation under ambient conditions, and the resulting assays generally fail to detect low‐abundance targets at the early stages of infection [14]. LFA is simple, rapid, and inexpensive, but conventional versions employ gold nanoparticles as labels that generate colorimetric signals through plasmonic resonance, often resulting in limited sensitivity [15]. Culture‐based methods are considered the “gold standard” for pathogen isolation and drug susceptibility testing [16], but they are labor‐intensive, time‐consuming, and in some cases impractical, such as the influenza virus, which may take several days to propagate [17]. Lastly, histological and microscopic methods allow direct visualization of pathogens or disease‐associated features, but they generally offer only qualitative or low‐throughput results [18]. As infectious disease outbreaks continue to emerge year after year, there is a growing need for diagnostic and screening platforms that are not only rapid, reliable, and cost‐effective but also require minimal equipment and can be operated without highly trained personnel.

Nanozymes, broadly defined as nanomaterials with intrinsic enzyme‐like catalytic activities, have become an emerging class of artificial biocatalysts in recent years [19, 20, 21]. Among them, peroxidase‐mimicking nanozymes (PMNs) represent an important subset that replicates the catalytic function of HRP and other natural peroxidases. Since the discovery of iron oxide (Fe_3_O_4_) nanoparticles with peroxidase‐like activity in 2007 [22], research and development in this field have expanded rapidly. A wide range of nanomaterials, including metals, metal compounds, carbonaceous nanostructures, and metal–organic framework (MOF)‐based nanostructures, have since been reported [23, 24, 25]. These materials have found applications in biomedicine, biosensing, food safety, agriculture, and environmental remediation [26, 27, 28, 29]. Among these diverse applications, their role as HRP substitutes in bioassay is particularly impactful. In a typical assay, PMNs are functionalized with bioreceptors (e.g., antibodies and aptamers) that specifically recognize target analytes. After binding and removal of unbound components, the amount of PMNs retained at the capture site is proportional to the analyte concentration. Upon subsequent addition of H_2_O_2_ and an appropriate substrate, the bound PMNs catalyze substrate oxidation to generate measurable signals that scale with the analyte amount [30]. Depending on the detection modality, different types of substrates can be employed. Chromogenic substrates such as 3,3′,5,5′‐tetramethylbenzidine (TMB), 2,2′‐azino‐bis(3‐ethylbenzothiazoline‐6‐sulfonic acid) (ABTS), 3,3′‐diaminobenzidine (DAB), *o*‐phenylenediamine (OPD), and 3‐amino‐9‐ethylcarbazole (AEC) yield distinct color changes upon oxidation [31]. Fluorogenic substrates such as 10‐acetyl‐3,7‐dihydroxyphenoxazine (Amplex Red) yield fluorescence upon oxidation to resorufin [32]. Chemiluminescent substrates such as luminol emit light as a result of oxidative reactions [33]. The diversity of these substrate systems provides versatile signal outputs, allowing PMNs to be flexibly integrated into a wide range of bioassays.

These PMN‐based bioassays are particularly attractive for infectious disease screening and diagnostics at the point‐of‐care (POC), owing to several unique advantages over HRP. First, they exhibit superior stability and robustness. During infection and inflammation, protease expression is markedly elevated [34]. Real clinical samples such as sputum, wound exudates, and blood‐derived samples (e.g., serum or plasma) are therefore protease‐rich environments that can lead to the degradation or deactivation of HRP, which may compromise assay sensitivity and reproducibility. In contrast, the inorganic or hybrid nature of PMNs renders their catalytic architectures intrinsically more resistant to proteolytic degradation, while surface modification with antifouling polymers (e.g., PEG) further enhances stability by minimizing nonspecific protein adsorption and enzymatic interference in complex clinical matrices [35]. Their robustness also extends to portable diagnostic devices such as flexible electrochemical sensors, where PMNs can withstand repeated mechanical stress (e.g., bending or stretching), ensuring reliable long‐term or continuous monitoring under ambient and otherwise harsh conditions. This enhanced robustness is especially relevant for neglected tropical infectious diseases such as *Chagas* disease, where serological testing is frequently performed in resource‐limited settings and where high temperatures, humidity, and protease‐rich blood samples can diminish the stability and performance of HRP‐based assays [36]. Second, their catalytic efficiency can be modulated to reach levels that are comparable to, or in some cases exceed, that of HRP, as reflected by the apparent catalytic constant (*K*_cat_, see Section 2.1 for details) [37]. When integrated into bioassays, this enhanced catalytic activity enables limits of detection (LOD) far below those attainable with protein enzymes, a critical feature for the diagnosis and screening of infectious diseases characterized by low biomarker concentrations at the early and chronic stages (e.g., HIV [38], Zika [39], Ebola [40], *Chagas* disease [7], and malaria [41]). Third, PMNs can be manufactured at low cost and transported or stored without stringent requirements. Large‐scale screening efforts for infectious diseases require significant quantities of catalytic reporters, and many PMN‐based reporters can be produced more economically than high‐purity, diagnostic‐grade HRP. They also eliminate the need for cold‐chain transport or storage, making them particularly suitable for outbreak scenarios in underdeveloped regions with limited infrastructure. Finally, PMNs offer multimodality. They can be engineered to integrate additional modalities such as photothermal, plasmonic, surface‐enhanced Raman scattering (SERS), fluorescence, and electromagnetic responsiveness [42, 43, 44]. These hybrid systems enable synergistic signal amplification while enhancing diagnostic reliability through internal validation across multiple detection modules.

**FIGURE 1:**
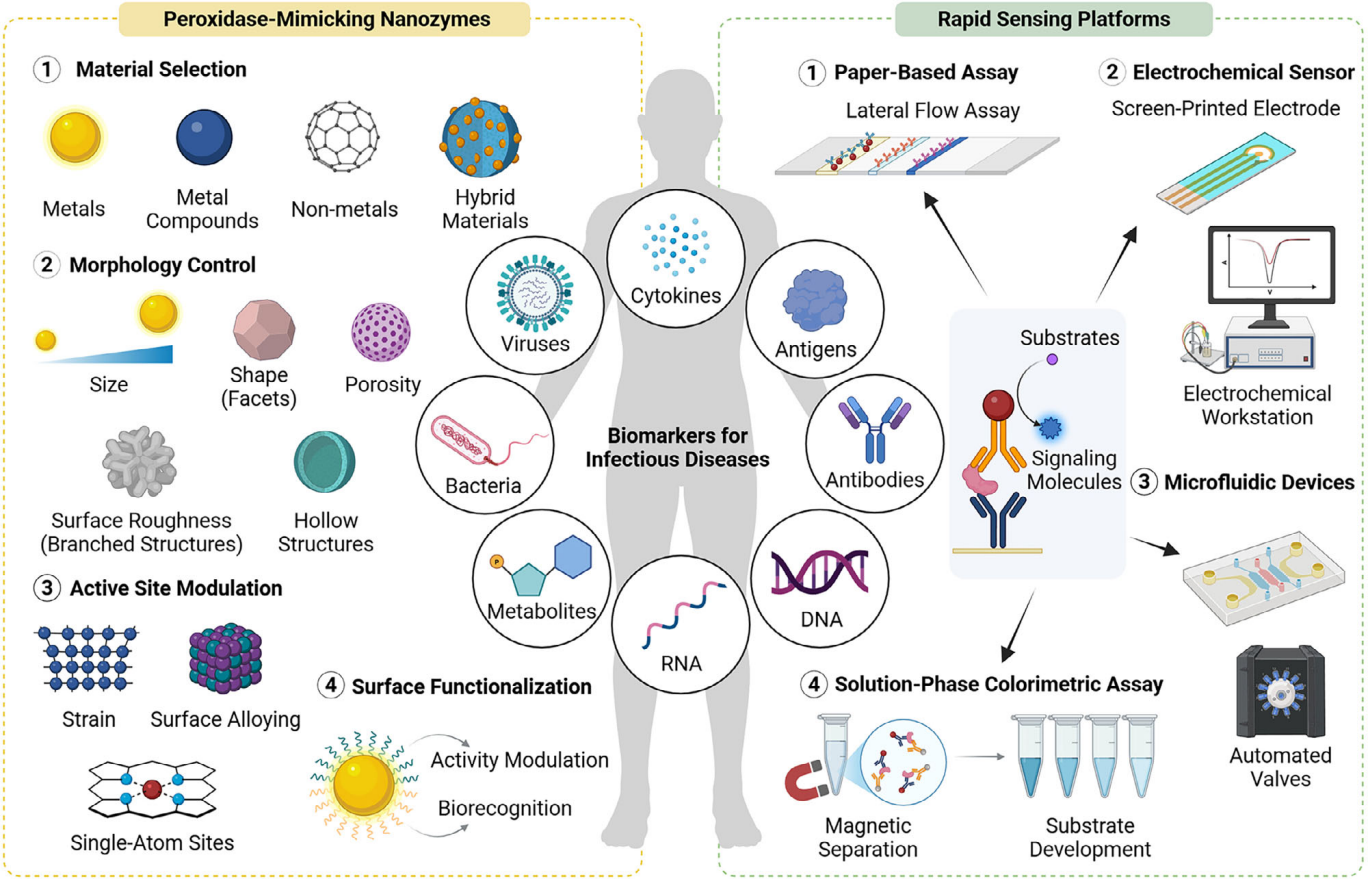
Schematic illustration of using peroxidase‐mimicking nanozymes (PMNs) for rapid detection of infectious disease‐relevant biomarkers. The left panel depicts the rational design of PMNs through material selection, morphology control, active site modulation, and surface functionalization. The right panel illustrates how these PMNs are incorporated into a variety of rapid sensing platforms to achieve sensitive and timely detection, including lateral flow assays, electrochemical sensors, microfluidic devices, and solution‐phase colorimetric assays. Created with BioRender.com.

This review provides a comprehensive overview of recent advances in this emerging field and identifies key factors that could accelerate the transition of PMNs into practical applications for infectious disease detection (Figure 1). We begin by outlining the fundamental design principles and optimization strategies for PMNs, followed by an examination of current solution‐phase synthetic approaches. Next, we summarize relevant biomarkers for infectious disease detection and describe how PMNs are integrated into various rapid biosensing platforms. We then discuss the cost‐effectiveness of PMNs and their broader societal impact. Finally, we conclude with an assessment of current challenges and future opportunities that will shape the continued development of this field and its associated technologies.

## 2 Rational Design of PMNs

The catalytic and other physicochemical properties of PMNs are critical for achieving sensitive detection and enabling additional functionalities within biosensing platforms. These properties are governed by factors such as elemental composition, morphology‐dependent accessibility of catalytic surfaces, the electronic structure of active sites, and the surface ligands that regulate interactions with substrates and reaction intermediates. In the following discussion, we examine how these design parameters can be rationally controlled and how they collectively influence the catalytic behavior and overall assay performance of PMNs.

### 2.1 Material Selection

Selecting appropriate materials represents the first step in the rational design of PMNs, as the elemental composition predominantly governs their intrinsic peroxidase‐mimicking activity and structural stability. Natural peroxidases, such as HRP, feature active centers composed of iron coordinated within a heme prosthetic group. In contrast, artificial enzyme mimics are not constrained by the elemental abundance in nature, and cytotoxicity is generally not a major concern for in vitro diagnostic applications. Consequently, a wide range of nanomaterials, including metals, metal compounds, porous framework materials, and carbonaceous structures, have been explored as PMNs in bioassays. It is noteworthy that the catalytic mechanism of PMNs can differ substantially from those of natural peroxidases, and the dominant pathways can also vary among different material classes. These catalytic behaviors are influenced not only by elemental composition but also by surface chemistry, electronic structure, and the reaction microenvironment [45]. Nevertheless, in diagnostic assays where the signal intensity primarily reflects the overall peroxidase‐like catalytic efficiency of the PMNs under optimized conditions, differences in the detailed reaction pathway generally have limited influence on the final signal output. Therefore, specific catalytic mechanisms are beyond the scope of this discussion.

For clarity, it should also be noted that the apparent catalytic constant (*K*_cat_) in this Review refers to an apparent parameter describing the peroxidase‐like catalytic efficiency of PMNs, defined as the maximum number of catalytic reaction products generated per second per single catalytic entity. While the question of how to properly compare catalytic activities of nanozymes has been discussed in the previous literature [46, 47], no unified normalization standard has yet been established. In this Review, to facilitate comparison across different PMN systems, we recommend the following normalization conventions: when discrete nanoparticles can be reasonably defined, *K*_cat_ values are normalized on a per‐particle basis; [22] For continuous or non‐discrete material architectures (e.g., layered structures, interconnected networks, or highly porous frameworks), where defining an individual particle is not physically meaningful, normalization by material amount (per unit mass or volume) is adopted. In addition, to enable comparison across materials with different sizes and morphologies, we use *K*_cat‐specific_, which is derived by further normalizing *K*_cat_ against the geometric surface area of the PMN material [48, 49].

Metal‐based PMNs are typically nanomaterials that employ noble metals as catalytic surfaces. Among them, platinum‐group metals (Ru, Rh, Pd, Os, Ir, and Pt) are particularly favored owing to their outstanding peroxidase‐like catalytic efficiency and superior stability [37]. For example, Pd–Ir core–shell nanocubes exhibited a *K*_cat_ at the level of 10^6^ s^−1^, approximately three orders of magnitude higher than that of HRP (10^3^ s^−1^). In addition to their remarkable catalytic efficiency, these nanocubes demonstrated excellent thermal and chemical stability, retaining activity comparable to that under standard assay conditions (22°C, pH 4) even after exposure to highly acidic (pH 0) and alkaline (pH 12) environments or extreme thermal treatment at 200°C [50]. Notably, nanozymes made of some noble metals (e.g., Pt and Au) possess both peroxidase‐ and oxidase‐like activities even under acidic conditions. This means that, in assays, they can catalyze substrate oxidation not only through H_2_O_2_ but also via dissolved O_2_, which may introduce high background or false‐positive signals. To address this issue, osmium (Os) nanoparticles offer an effective solution, as they display minimal oxidase‐like activity while maintaining high peroxidase‐like activity. This selectivity originates from their unique surface electronic configuration, where weak O_2_ adsorption and high O─O bond dissociation barriers effectively suppress unwanted oxidase‐like catalytic activities [51].

Metal compound‐based nanostructures represent another important class of PMNs. Typical nanostructures include metal oxides (e.g., Fe_3_O_4_, CeO_2_) [22, 52], metal sulfides (e.g., FeS_2_) [53], spinel oxides (e.g., CoFe_2_O_4_) [54], perovskite oxides (e.g., LaFeO_3_) [55], and metal coordination compounds (e.g., Prussian Blue) [56]. These compounds are generally low‐cost but often exhibit limited stability compared with noble metal‐based PMNs. Among them, Fe_3_O_4_ nanoparticle, as a typical metal oxide, PMN exhibits a *K*_cat_ comparable to that of HRP, but its catalytic durability is limited. This is because Fe_3_O_4_ is prone to structural and compositional degradation during catalysis. Under acidic conditions (pH ≤ 4), surface Fe^2+^/Fe^3+^ ions are gradually leached [57], while during repeated catalytic cycles in bioassays, lattice Fe^2+^ is progressively oxidized, leading to a phase transformation from Fe_3_O_4_ to γ‐Fe_2_O_3_ accompanied by depleted peroxidase‐like activity [58]. Instead, metal carbides (e.g., WC) [59] and metal nitrides (e.g., Co_4_N) [60] have attracted attention as more robust metal compound‐based PMNs. These materials combine high catalytic efficiency with remarkable tolerance to temperature, pH, and salinity, providing superior stability and reusability relative to conventional metal oxide PMNs.

Porous framework materials such as MOFs and covalent‐organic frameworks (COFs) have recently emerged as promising PMNs. These crystalline materials are constructed via the self‐assembly of metal ions or metal nanoclusters with organic ligands, featuring high surface areas and ordered pore structures that render their catalytic sites readily accessible. However, their coordination networks are often fragile and prone to compositional and structural collapse in aqueous environments [61]. To address these limitations, one‐step pyrolysis can transform the frameworks into carbon‐based MOF derivatives with enhanced robustness and conductivity, while preserving or reconstructing their atomic‐level metal–ligand coordination. This approach also allows precise control over the active sites and their coordination environments, yielding heme‐inspired structures similar to those found in natural peroxidases. For example, pyrolysis of a ZIF‐8 precursor forms Fe–N_4_ single‐atom sites through a gas‐phase migration process, in which volatile Fe(CNx) species are transported and trapped by N‐rich porous carbon. The resulting Fe single‐atom catalysts display enhanced peroxidase‐like activity, exceeding that of Fe_3_O_4_ PMNs [62].

Instead of using metals as active centers, carbon‐based PMNs, such as graphene oxide, carbon nanospheres, carbon nanotubes, and carbon dots, have also been widely explored [63, 64]. Although their catalytic efficiency is generally lower than that of metal‐based nanozymes, these materials are inexpensive, chemically stable, and inherently biocompatible owing to their nonmetallic composition, which minimizes the risk of denaturing recognition elements in bioassays. Their biocompatibility has also enabled innovative sensing designs that integrate in vivo enrichment with in vitro readout. For example, carbon quantum dots (CQDs) have been incorporated into an aptamer–DNAzyme–nanozyme sensor for Alzheimer's disease detection: the disease biomarker activates the DNAzyme in vivo, triggering the release of CQDs, which are then filtered by the kidneys and collected in urine. The peroxidase‐like activity of the CQDs subsequently generates a measurable colorimetric signal upon addition of TMB and H_2_O_2_. The safety of such CQD‐based nanozymes has been verified by 3‐(4,5‐dimethylthiazol‐2‐yl)‐2,5‐diphenyltetrazolium bromide (MTT) cytotoxicity assays, stable body weight in mice, and the absence of histological or hematological abnormalities [65]. In addition, carbon‐based PMNs can be synthesized with abundant surface functional groups that facilitate substrate adsorption and biomolecule conjugation. Their defect‐rich structures are well suited for heteroatom doping (e.g., O, P, N, S, or metal atoms) to further enhance catalytic efficiency [63]. Collectively, these features make carbon‐based PMNs promising candidates for biosensing applications.

**FIGURE 2:**
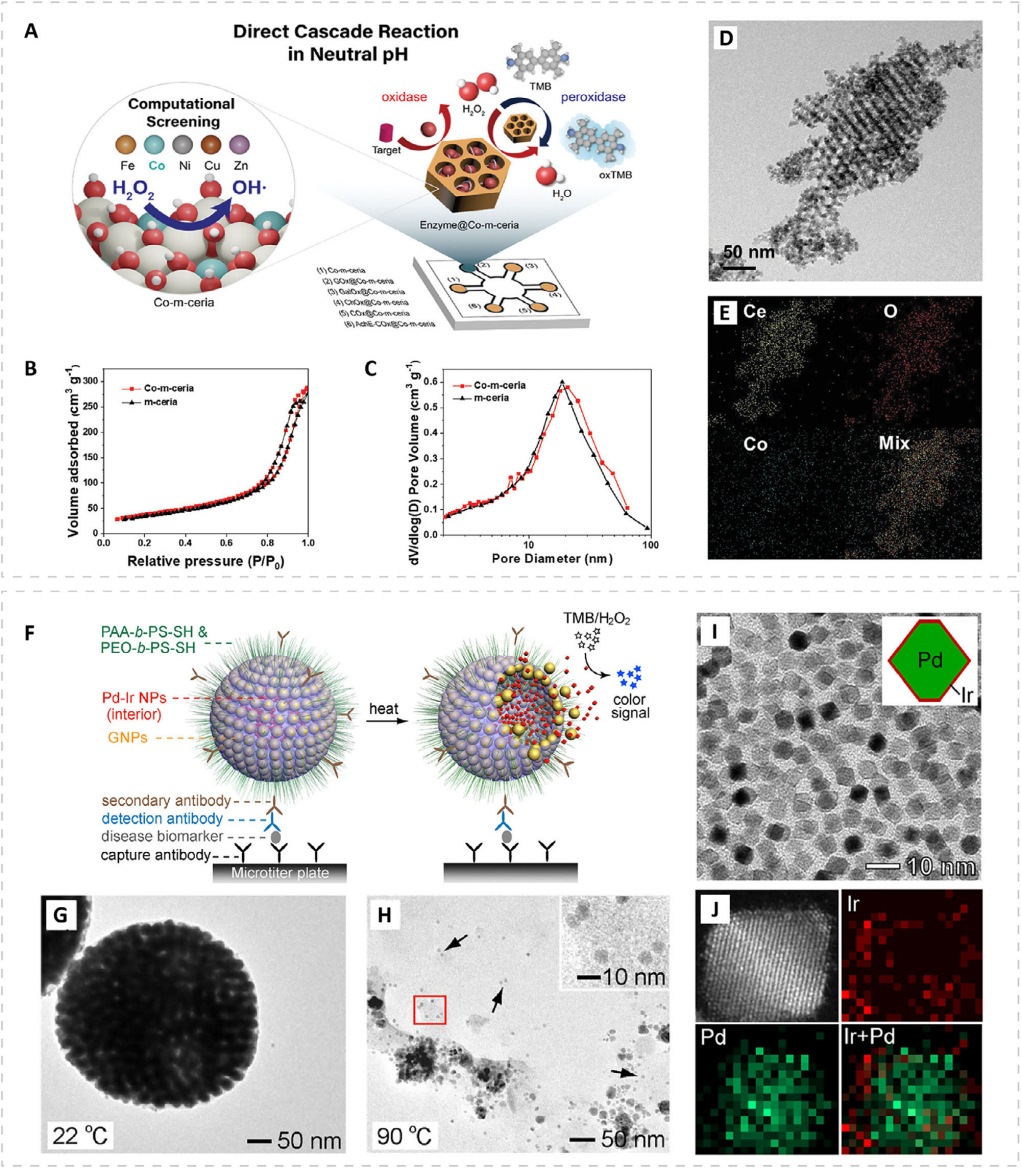
Examples of hybrid PMNs. (A–E) Hybrid PMNs with cascade design. (A) Schematics showing indirect metabolite detection using oxidase‐immobilized Co‐doped mesoporous ceria PMNs (Enzyme@Co‐m‐ceria), where oxidase‐catalyzed reactions generate H_2_O_2_ for subsequent peroxidase‐like readout. (B) N_2_ adsorption–desorption isotherms of Co‐m‐ceria and m‐ceria, and (C) corresponding pore size distributions calculated from the adsorption curves of the isotherms using the Barrett–Joyner–Halenda method. (D) Transmission electron microscopy (TEM) image and (E) elemental mapping of Co‐m‐ceria. Reproduced with permission [66]. Copyright 2022, Wiley. (F–J) Hybrid PMNs with signal amplification design. (F) Schematics showing heat‐responsive gold vesicles with encapsulated small Pd–Ir nanoparticles (Pd–Ir NPs@GVs), which release active PMNs upon heating. (G,H) Representative TEM images of Pd–Ir NPs@GVs after 1 h of heat treatment at (G) 22°C and (H) 90°C. (I) TEM image and (J) elemental mapping of the small Pd–Ir nanoparticles. Reproduced with permission [67]. Copyright 2017, American Chemical Society.

Beyond single‐component systems, multiple types of the above materials, along with natural enzymes, can be integrated into one nanostructure to form hybrid nanozymes. These hybrid systems can be designed in a cascade configuration to detect metabolites or biomarkers whose levels fluctuate during infection or inflammatory responses but cannot be directly recognized by PMNs alone (Figure 2A–E). For example, oxidase enzymes have been immobilized within the mesopores of Co‐doped mesoporous ceria (Co‐m‐ceria), where they catalyze the oxidation of their corresponding metabolites and simultaneously generate H_2_O_2_. Then, the generated H_2_O_2_ is detected through the peroxidase‐like activity of the Co‐m‐ceria framework by simply adding TMB, enabling efficient cascade detection under near‐neutral conditions [66]. Hybrid nanozyme architectures can also be engineered for signal amplification (Figure 2F–J). For example, small Pd–Ir nanoparticles encapsulated within gold vesicles (Pd–Ir NPs@GVs), in which mild heating triggers vesicle disassembly and releases highly active Pd–Ir nanoparticles with superior peroxidase‐like activity. When employed as catalytic reporters in ELISA, the heat‐triggered release amplifies colorimetric signals, offering high sensitivity for biomarker detection [67].

When selecting PMNs for bioassays, it is important to consider that some nanomaterials can be designed to provide multimodality, offering additional optical, thermal, or electrical signals beyond catalysis within a single biosensing platform. First, such integrated physicochemical features expand detection options and enable internal validation. For example, urchin‐shaped Au‐Ag@Pt nanoparticles simultaneously exhibit plasmonic, photothermal, SERS, and catalytic activities. When used as labels in the LFA platform, this multimodality allows sensitive detection through multiple signal channels. Notably, the SERS mode provides characteristic Raman fingerprints that enable specific bacterial differentiation by recording and identifying unique spectra for different bacterial species [44].

Second, beyond multimodality derived directly from intrinsic physicochemical properties, electroactive nanostructures can be exploited to enable electrochemical readouts while maintaining peroxidase‐like activity for colorimetric detection. Prussian blue nanoparticles (PBNPs) provide a representative example, in which the cyanide‐bridged Fe^2+^/Fe^3+^ coordination framework endows PBNPs with peroxidase‐like catalytic activity, while the large lattice cavities allow entrapment of alkali metal ions (e.g., Na^+^ and K^+^), which have been reported to enhance electron transport in electrocatalytic systems [68]. In electrochemical sensing, PBNPs function as electroactive labels in sandwich‐type immunoassays [69], where formation of the immunocomplex leads to accumulation of PBNP‐labeled detection antibodies on the electrode surface and generates enhanced current signals arising from the intrinsic redox activity of surface‐confined PBNPs. Concurrently, oxidation of TMB by the same nanozyme system produces a visible colorimetric response.

Third, multimodality can be implemented through mechanistically signal switching triggered by molecular recognition, rather than by simply adding another detection channel. In an aptamer/GCN dual‐mode system [70], the 6‐carboxyfluorescein (FAM)‐labeled single‐stranded (ss) DNA aptamer adsorbs onto graphitic carbon nitride (GCN) primarily through π–π interactions. This adsorption produces two coupled effects: (i) fluorescence is quenched because short‐range photoinduced electron transfer (PET) occurs from excited FAM to the electron‐accepting GCN surface, and (ii) the peroxidase‐like colorimetric signal is amplified because the negatively charged aptamer layer enhances electrostatic attraction and local enrichment of the positively charged TMB substrate, thereby increasing the apparent TMB oxidation rate. When the target bacterium (in this case, *Salmonella Typhimurium*, a representative foodborne infectious pathogen) is present, the aptamer preferentially detaches from GCN to bind the bacterial surface. This recognition event simultaneously relieves PET quenching and restores fluorescence (“signal‐on” fluorescence) and removes the aptamer‐driven TMB enrichment effect, returning GCN toward its intrinsic weaker peroxidase‐like activity and reducing the blue oxTMB color (“signal‐off” colorimetry). In this way, the same recognition event enforces mutually consistent changes in two orthogonal outputs, improving confidence and reducing ambiguous interpretation. This method achieves LOD for *Salmonella* as low as 8 and 3 CFU mL^−1^ when using colorimetric and fluorometric readouts, respectively.

In addition, PMNs can be engineered to possess magnetic responsiveness, which is critical for analyte enrichment from complex biological matrices and for minimizing false‐positive signals caused by non‐specific interactions. A representative example is the magnetic virus‐like nanozyme Fe–Pt_20_–Au@Pt_5_, whose Fe_3_O_4_ core provides rapid magnetic capture while its Pt/Au@Pt shell generates a dense array of catalytic hotspots. This dual functionality enables direct pathogen detection in stool samples by combining efficient magnetic extraction with catalytic signal amplification. Note that stool is widely regarded as one of the most challenging clinical matrices because of its high complexity [71].

When considering multimodality at the material level, it is important to recognize that the practical value of PMN‐based platforms depends on selecting materials whose signal outputs can operate compatibly and reliably within an integrated sensing system. In practice, different materials that support multiple signal modalities often differ substantially in amplification factors, dynamic range, and noise characteristics, and poorly matched readouts can become uneven in reliability or unnecessarily complicate data interpretation rather than improve confidence. In addition, the chemical and physical environments required for peroxidase‐like colorimetric reactions may interfere with other signal channels: chromogenic substrates and their oxidation products can introduce optical background or surface fouling, while H_2_O_2_ exposure and photothermal operation may induce chemical etching, surface reconstruction, or ligand degradation, thereby altering particle morphology and associated optical properties as well as subsequent peroxidase activity [31]. Finally, certain modalities impose constraints on colloidal stability, as exemplified by magnetic nanozymes that can promote particle aggregation and reduce catalytic accessibility if not properly stabilized.

### 2.2 Morphology Control

The catalytic behavior of nanozymes is largely determined by the accessible surface. Accordingly, both the accessible surface area and the types of exposed crystal facets strongly influence the catalytic efficiency of PMNs. These characteristics can be tuned through morphological control, including the adjustment of particle size, shape, hollowness, porosity, and surface roughness.

A straightforward approach is to tune the size of the nanozyme. For example, in Pd–Ir core–shell PMNs, the catalytic efficiency quantified by the *K*_cat_ value increased as the particle size increased from 3.3 nm to 13 nm, while the area‐specific catalytic activities (*K*_cat‐specific_) remained similar, indicating that the improvement in catalytic performance mainly arises from the enlarged accessible catalytic surface [49]. Interestingly, when these nanozymes were used as reporters in ELISA, smaller nanoparticles produced lower LOD. This trend could be explained by the fact that smaller nanoparticles have higher diffusivities and reduced steric hindrance, which makes them easy to bind to analytes through antibody–antigen interaction.

**FIGURE 3:**
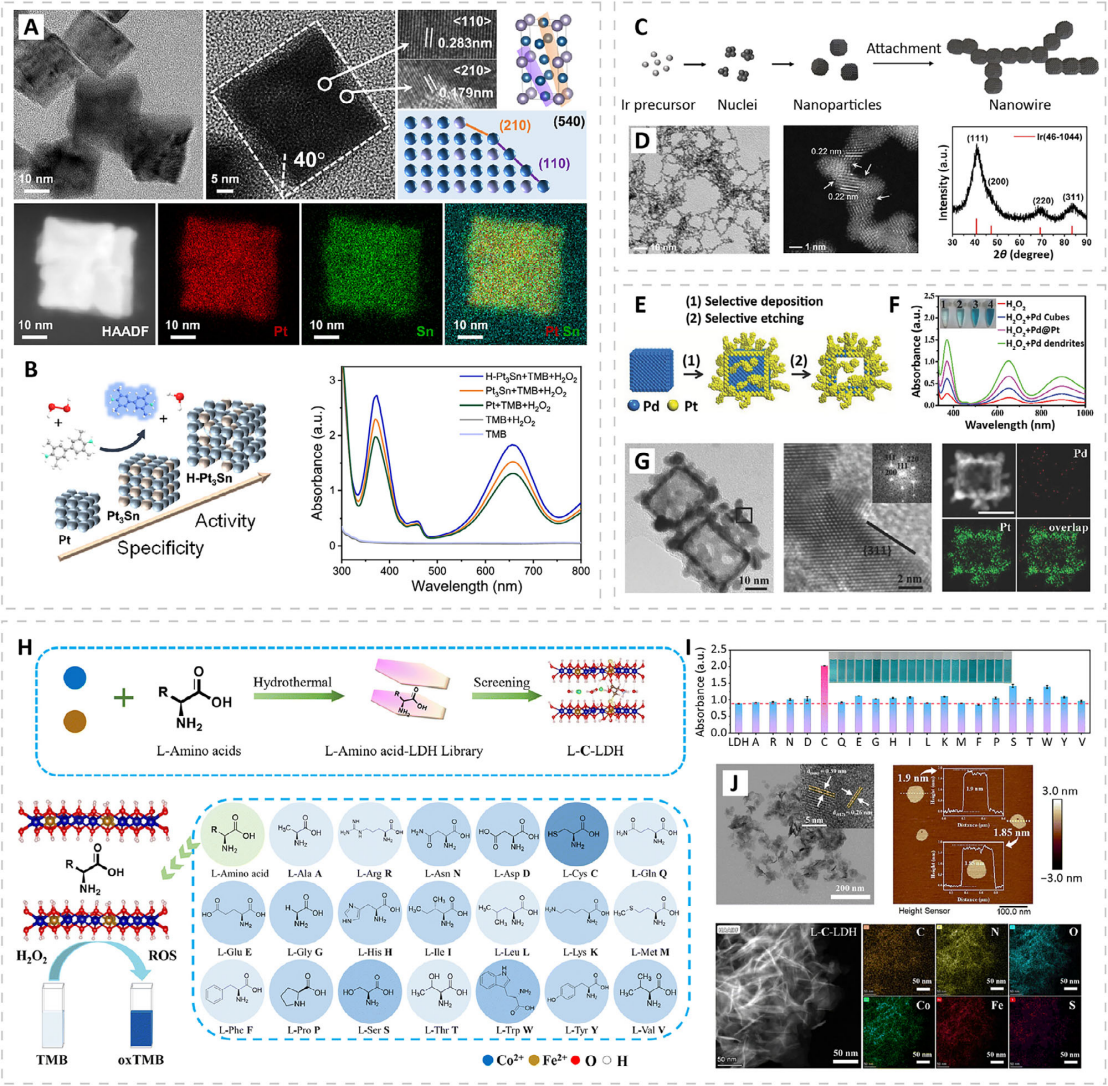
Examples of morphology control in PMNs. (A,B) High‐index facets. (A) TEM images, elemental mappings, and structural models showing the (540) terrace step features of high‐indexed intermetallic Pt_3_Sn (H‐Pt_3_Sn). (B) Schematic illustration and absorbance spectra comparing the peroxidase‐like activities of pure Pt, Pt_3_Sn, and H‐Pt_3_Sn nanozymes. Reproduced with permission [74]. Copyright 2022, American Chemical Society. (C,D) Open or anisotropic structures. (C) Schematics showing the formation process and structure of Ir nanowires. (D) TEM images and x‐ray diffraction (XRD) pattern of the Ir nanowires. Reproduced with permission [76]. Copyright 2023, American Chemical Society. (E–G) Dendritic surface structures. (E) Schematics showing the formation of Pt hollow nanodendrites through selective deposition followed by selective etching. (F) Absorbance spectra comparing the peroxidase‐like activities of Pd cubes, Pd@Pt core‐frame nanodendrites, and Pt hollow nanodendrites. (G) TEM images and elemental mapping of the Pt hollow nanodendrites. Reproduced with permission [78]. Copyright 2018, Wiley. (H–J) Two‐dimensional PMNs. (H) Schematics showing the synthesis of amino acid‐intercalated CoFe‐layered double hydroxides (L‐AA‐LDH) and the screening of their peroxidase‐like activity with different amino acids. (I) Histogram comparing the absorbance at 652 nm for the L‐AA‐LDHs library generated using various amino acids, with L‐cysteine‐intercalated LDH (L‐C‐LDH) exhibiting the highest activity. (J) TEM image, atomic force microscopy (AFM) image, and elemental mapping of the L‐C‐LDH nanozyme. Reproduced under license CC BY‐NC 3.0.

Shape is another important factor governing the catalytic activity of PMNs, and its influence can be understood from two aspects. On one hand, the shape of a nanozyme determines the arrangement and coordination of surface atoms, which modulate how reactants bind and how reaction intermediates are adsorbed, thereby affecting catalytic efficiency. These effects arise from two mechanisms: (i) facets with different atomic geometries exhibit distinct reaction energetics. For example, Pd nanocubes with exposed (100) facets show higher peroxidase‐like activity than Pd octahedra with (111) facets. This is because the homolytic dissociation of H_2_O_2_ species, which is the rate‐determining step in TMB oxidation, proceeds more easily on the (100) surface as it orients the ∙OH intermediate in a configuration that lowers the H_2_O_2_ dissociation barrier; [72] and (ii) high‐index facets that contain steps, ledges, and kinks further enhance activity by providing catalytically active low‐coordinated surface atoms along with a higher density of surface defects [73]. For example, high‐indexed intermetallic Pt_3_Sn (H‐Pt_3_Sn) nanozymes with (540) facets display superior peroxidase‐like activity and high pathway specificity compared to regular Pt_3_Sn and pure Pt nanozymes (Figure 3A,B) [74]. On the other hand, shape also influences the total accessible catalytic surface, which directly determines the number of active sites available for reaction. This effect is particularly evident in open or anisotropic structures [75, 76]. For example, Ir‐ and Pt‐based nanowires possess narrow diameters and high aspect ratios that enlarge the total accessible catalytic surface (Figure 3C,D), resulting in higher peroxidase‐like activities than their spherical counterparts [76].

Other structural parameters, in addition to size and shape, can also be modulated to further enhance peroxidase‐like activity. Nanoparticles with dendritic surface structures provide a representative example, as the branched morphology exposes abundant catalytically active sites, many of which lie on high‐index facets [77, 78, 79]. For instance, Pt hollow nanodendrites exhibit substantially higher peroxidase‐like activity than Pd@Pt core–frame nanodendrites and Pd cubes because their hollow interiors are open and accessible (Figure 3E–G), while their dendritic branches expose a large number of highly active sites located on high‐index facets such as (311) [78]. Moreover, nanoparticles with mesoporous architectures (e.g., hollow CuO spheres or mesoporous Fe_2_O_3_) [80, 81] also show excellent peroxidase‐mimicking performance. In addition to their increased surface area, these materials present interconnected mesoporous channels that facilitate substrate and product diffusion, thereby accelerating catalytic reactions. This type of porous structure can also accommodate guest species such as enzymes, metal nanoparticles, and reporter molecules. For example, Co‐doped mesoporous ceria frameworks allow the loading of oxidase enzymes to enable cascade reactions (Figure 2A) [66].

It is worth mentioning that 2D materials have recently attracted considerable attention as PMNs. Their ultrathin thickness ensures that nearly all catalytic centers reside at or near the surface, providing extensive accessible sites that facilitate substrate interaction and accelerate catalytic turnover. In addition, their in‐plane atomic arrangement allows precise tuning of active‐site composition and the surrounding electronic environment through functionalization or defect engineering. Representative 2D PMNs include graphene oxide/reduced graphene oxide nanosheets, graphitic carbon nitride (g‐C_3_N_4_), 2D MOFs, transition‐metal dichalcogenides, layered double hydroxides (LDHs), and layered transition‐metal oxides [82]. In a recent design, the peroxidase‐like activity of CoFe‐LDH nanosheets was markedly enhanced by intercalating amino acids to modulate the catalytic microenvironment (Figure 3H–J) [83]. The amino acids enlarge the interlayer spacing to improve substrate diffusion and simultaneously introduce abundant oxygen vacancies, which facilitate H_2_O_2_ activation by lowering the barrier for O─O bond cleavage and promoting electron transfer during substrate oxidation. Among all the amino acids examined, L‐cysteine provided the most significant enhancement owing to its thiol functional group, which strongly coordinates with metal centers and effectively modulates the local catalytic environment. Besides direct activity modulation, 2D materials are also widely used as supports to carry metal nanoparticles [84, 85]. Their layered structures prevent nanoparticle aggregation, improve aqueous dispersion, and promote particle‐support interfacial electron transfer, which enhances the overall catalytic performance of the resulting composite. For example, Pt and Cu nanoparticles can be deposited in situ onto Ti_3_C_2_T_x_ MXene nanosheets to form MXene@PtCu PMNs, which has been applied as catalytic labels in a LFA for melioidosis [85]. In this design, the high surface area and hydrophilicity of Ti_3_C_2_T_x_ promote uniform nanoparticle loading and stable dispersion. The strong interaction between Pt and Cu nanoparticles provides synergistic catalytic enhancement, while the MXene support further contributes by providing a conductive 2D scaffold that accelerates electron transfer across the metal–support interfaces during substrate oxidation. These features together endow MXene@PtCu with high peroxidase‐like activity and enable efficient conjugation with nucleic acid probes. When incorporated into a catalytic hairpin assembly‐assisted LFA, the nanozyme labels produced markedly amplified colorimetric signals, allowing ultrasensitive detection of the *B. pseudomallei* sRNA biomarker BprsO with a LOD of 1.26 fM.

### 2.3 Active Site Modulation

Active sites lie at the heart of both natural enzymes and PMNs. The key difference is that, in PMNs, these sites can be intentionally engineered through advanced nano‐synthetic approaches, allowing controlled modulation of their electronic structures.

**FIGURE 4:**
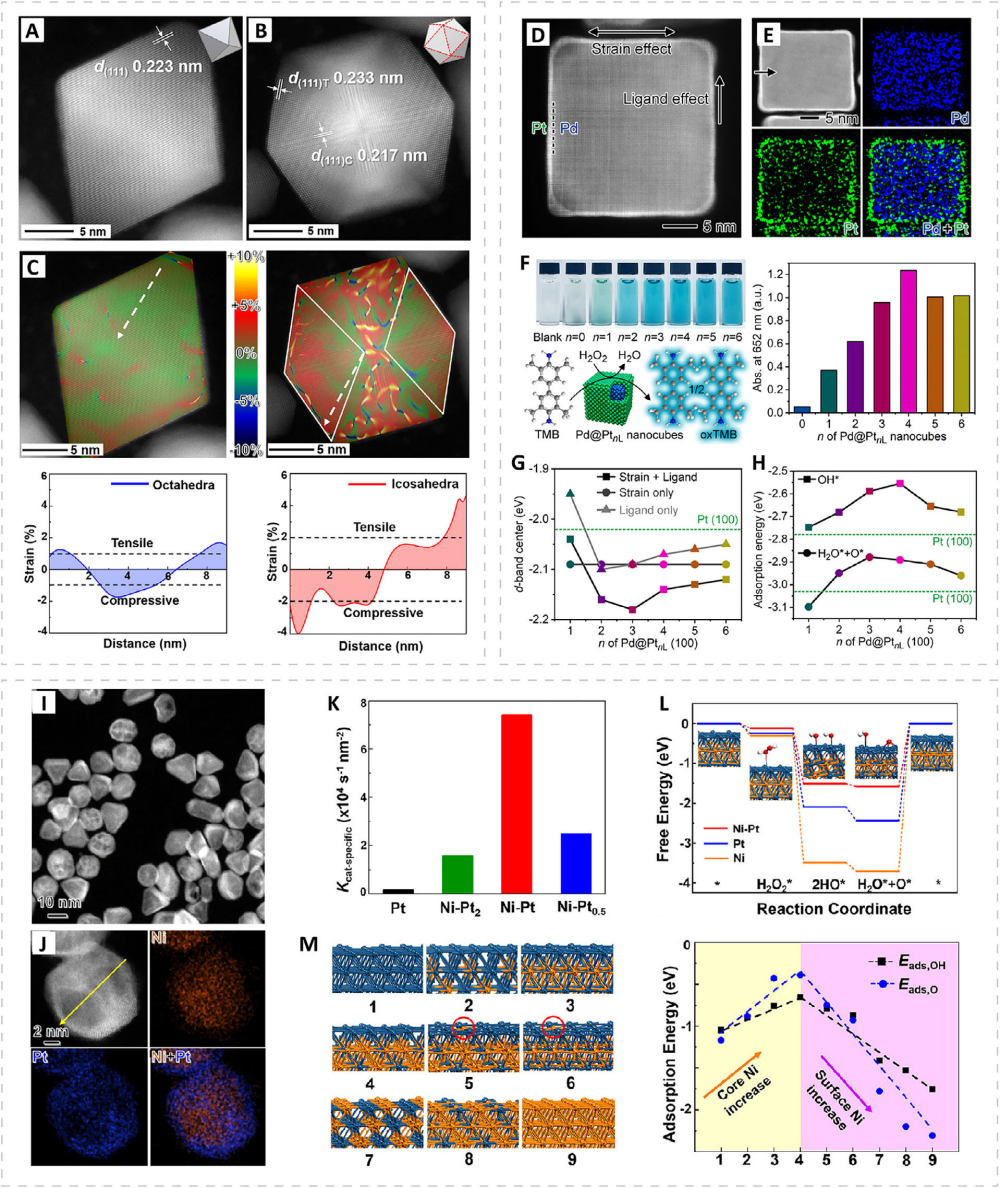
Active‐site modulation through surface strain and alloying in PMNs. (A–C) Surface strain arising from particle geometry. (A,B) Atomic‐resolution HAADF‐STEM images of individual (A) Pd octahedra and (B) Pd icosahedra. (C) Corresponding surface strain mapping and line‐profile analyses obtained through geometric phase analysis. Reproduced with permission [86]. Copyright 2019, American Chemical Society. (D–F) Strain and ligand effects induced by lattice mismatch. (D) Atomic‐resolution HAADF‐STEM image of an individual Pd@Pt_4L_ nanocube illustrating combined strain and ligand effects, and (E) the corresponding elemental mapping. (F) Photographs and absorbance comparison at 652 nm of TMB–H_2_O_2_ solutions catalyzed by Pd@Pt*_n_*_L_ nanocubes. (G) DFT‐calculated *d*‐band centers of Pd@Pt*_n_*_L_ (100) surfaces showing contributions from strain, ligand, and combined effects. (H) DFT‐calculated adsorption energies of *OH and H_2_O* + O* on Pd@Pt*_n_*_L_ (100) surfaces. Reproduced with permission [87]. Copyright 2025, American Chemical Society. (I–M) Surface alloying. (I) HAADF‐STEM image of Ni–Pt nanoparticles and (J) elemental mapping of an individual particle. (K) Area‐specific catalytic efficiencies (*K*_cat‐specific_) of Ni–Pt nanoparticles with varying Ni/Pt ratios. (L) DFT‐calculated free‐energy diagrams for H_2_O_2_ decomposition and optimized adsorption configurations on Ni–Pt surfaces. (M) Slab models and corresponding adsorption energies for OH and O on Ni–Pt surfaces. Blue atoms represent Pt, and orange atoms represent Ni. Reproduced with permission [88]. Copyright 2021, American Chemical Society.

Introducing surface strain provides a means to modulate the electronic structure of PMNs by altering surface atomic distances, thereby adjusting the interaction of substrates or intermediates with catalytic surfaces. In our previous work, we systematically investigated the strain effect using Pd octahedra and icosahedra as model systems (Figure 4A–C) [86]. An atomic resolution high‐angle annular dark‐field scanning TEM (HAADF‐STEM) image revealed distinct twin boundaries on Pd icosahedra. Near these boundaries, the lattice spacing was narrowed, indicating compressive strain, whereas the central facets exhibited expanded spacing characteristics of tensile strain. In contrast, Pd octahedra displayed lattice spacings close to bulk Pd and thus showed negligible strain. This observation was further supported by geometric phase analysis, which revealed alternating tensile and compressive domains across the icosahedral surface. This intrinsic strain led to an approximately two‐fold enhancement in *K*_cat‐specific_ compared to Pd octahedra. Beyond generating strain intrinsically through nanoparticle shape, strain can also be introduced in a controlled manner by depositing an ultrathin metal shell onto a template with a different lattice constant. For example, conformally coating Pd nanocubes with atomic layers of Pt imposes compressive strain on the Pt shell due to lattice mismatch, while interfacial electron transfer introduces an additional ligand effect (Figure 4D–H) [87]. These two effects synergistically downshift the Pt *d*‐band center and weaken the adsorption of key intermediates (e.g., OH* and O*). As a result, the peroxidase‐like activity follows a shell‐thickness‐dependent volcano trend, with the Pd@Pt_4L_ (Pd core@Pt shell consisting of four atomic layers) nanocubes showing the highest activity.

Surface alloying represents another effective strategy for modulating the electronic structure of PMNs. For example, Ni–Pt alloyed nanoparticles can be synthesized with controlled surface composition to fine‐tune their electronic states (Figure 4I–M) [88]. A moderate amount of surface Ni weakens the adsorption of reaction intermediates and facilitates electron transfer to the TMB substrate, which leads to substantially enhanced peroxidase‐like activity compared with pure Pt nanozyme. As the Ni content continues to increase, however, the adsorption becomes too weak, and the activity declines, resulting in a volcano‐type dependence on the surface Ni composition. It is worth mentioning that in addition to improving catalytic activity, surface alloying can also enhance particle stability. A pure Ag surface is prone to leaching in the presence of H_2_O_2_ and is therefore not ideal for bioassays [89]. In contrast, Au@Ag‐Pt nanorattles formed through galvanic replacement reaction exhibit an Ag–Pt alloy shell that resists Ag dissolution and provides markedly improved thermal and chemical stability, while simultaneously offering much higher peroxidase‐like activity [90]. Beyond binary and ternary alloys, high‐entropy alloys (HEAs) extend this concept by mixing five or more elements into a single‐phase solid solution. This leads to lattice distortion and multisite *d‐*orbital coupling, which increases the electron abundance near the Fermi level through “self‐complementation” effect and enhances electron‐transfer efficiency at the catalytic interface through “cocktail” effect. Consequently, these electronic advantages lower the activation barrier for O─O bond cleavage in H_2_O_2_, leading to substantially higher peroxidase‐like activity [91, 92]. For example, PdMoPtCoNi high‐entropy nanoalloys exhibit strong *d*‐orbital overlap among Pd, Mo, Pt, Co, and Ni sites and show significantly higher peroxidase‐mimicking activity than their corresponding low‐entropy alloys (e.g., PdMoPt alloys) [92]. These HEA nanozymes were then integrated with a portable electronic readout device to enable sensitive POC digital detection of urinary glucose, sarcosine, and *Proteus mirabilis* (*P. mirabilis*, a Gram‐negative pathogen often associated with urinary tract infections).

**FIGURE 5:**
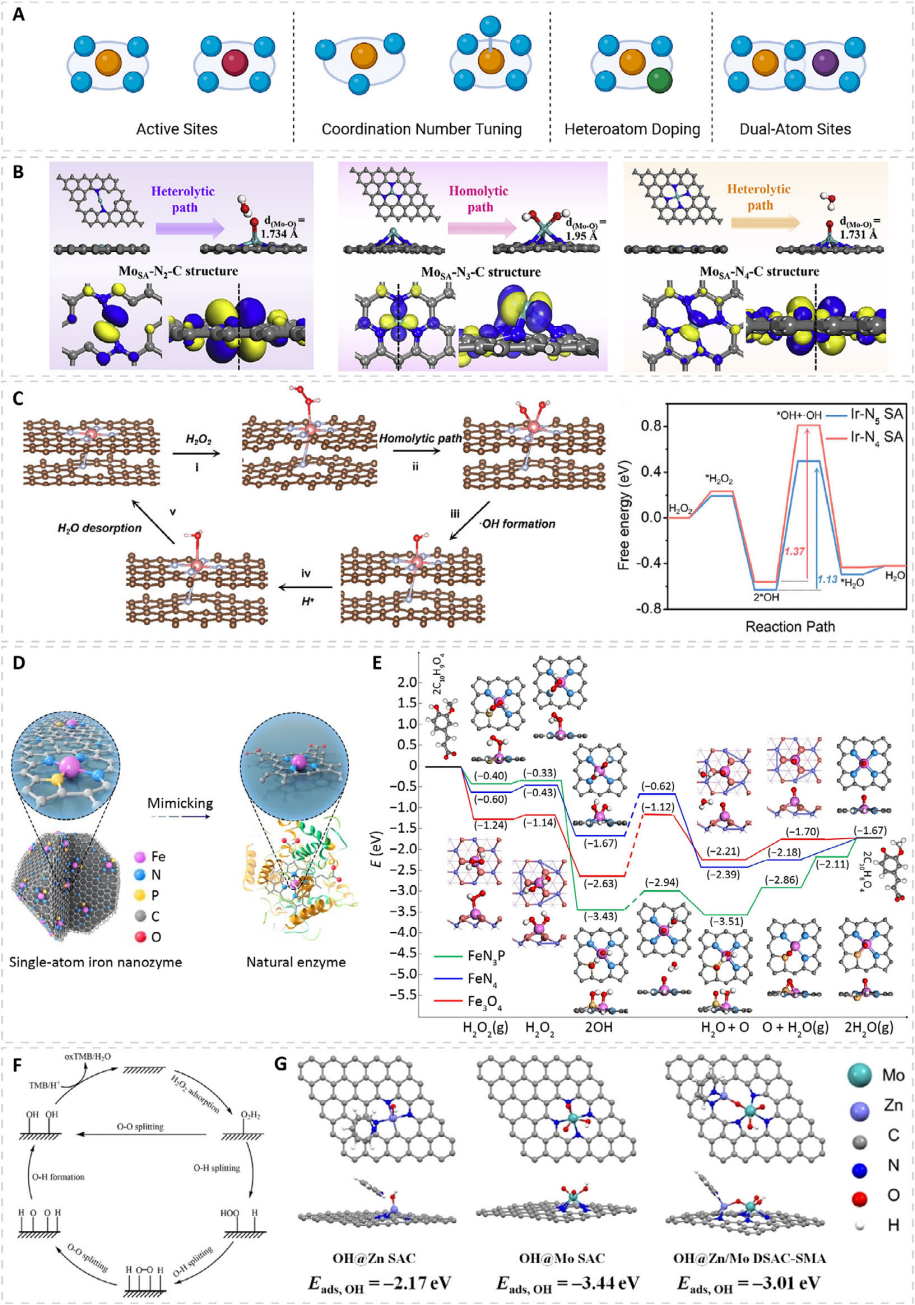
Active‐site modulation in single‐atom nanozymes (SAzymes). (A) Schematic showing strategies for tuning the active sites of SAzymes, including selecting the central metal site, adjusting coordination number, introducing heteroatom dopants, and constructing dual‐atom sites. Created with BioRender.com. (B) Removing coordination ligands. Models of Mo single‐atom sites with different coordination structures (MoSA–N_2_–C, MoSA–N_3_–C, and MoSA–N_4_–C) and their H_2_O_2_ activation pathways via hemolytic or heterolytic cleavage, along with HOMO distributions. Reproduced with permission [98]. Copyright 2020, Elsevier. (C) Introducing additional coordination ligands. Proposed reaction mechanism of Ir–N_5_ SAzymes during peroxidase‐like catalysis and free‐energy comparison between Ir–N_5_ and Ir–N_4_ SAzymes. Reproduced with permission [99]. Copyright 2023, Wiley. (D,E) Introducing heteroatom ligands. (D) Schematics comparing the Fe–N_3_P active site in SAzymes with the Fe–N_4_ site in natural horseradish peroxidase. (E) DFT‐calculated free‐energy diagrams comparing the peroxidase‐like catalytic pathways of FeN_3_P SAzyme, FeN_4_ SAzyme, and Fe_3_O_4_ nanozyme. Reproduced with permission [100]. Copyright 2021, Springer Nature Limited. (F,G) Dual‐metal‐site cooperation. (F) Proposed reaction pathways for peroxidase‐like nanozymes. (G) Computational models and optimized OH adsorption structures on Zn SAzymes, Mo SAzymes, and Zn/Mo dual‐site SAzymes, with corresponding OH adsorption energies (*E*_ads, OH_) listed at the bottom. Reproduced with permission [101]. Copyright 2022, Wiley.

Single‐atom nanozymes (SAzymes) provide one of the most structurally well‐defined platforms for active‐site engineering, as their atomically dispersed metal centers closely emulate the coordination structures of natural metalloenzymes (Figure 5A) [93, 94, 95, 96]. The typical SAzyme configuration consists of individual metal atoms anchored on nitrogen‐doped carbon frameworks, creating uniform geometric and electronic environments and enabling maximum atomic utilization. The design of carbon‐supported peroxidase‐mimicking SAzymes, therefore, begins with selecting the metal center. For example, a systematic comparative study of 20 M–N–C SAzymes with matched geometries revealed that different metal sites produce distinct reactivities. Among these SAzymes, Cu–N sites display the highest peroxidase‐like activity, whereas Co–N sites show the lowest [97]. Beyond the metal identity, the coordination environment, including the number, type, and geometry of coordination ligands, provides additional tunability to the single‐atom site. This can be introduced through several key aspects: (i) Coordination number. For example, in Mo–N_x_ single‐atom sites, the Mo–N_3_ configuration positions the Mo atom above the N_x_C plane, which improves the frontier‐orbital matching with H_2_O_2_ and favors a homolytic O─O cleavage pathway (Figure 5B). This accounts for the markedly higher peroxidase‐like activity of Mo–N_3_ relative to Mo–N_2_ and Mo–N_4_ [98]. Instead of removing ligands to expose the metal center, in Ir SAzymes, introducing an additional axial N ligand directly converts a planar Ir–N_4_ site into an Ir–N_5_ configuration with asymmetric charge distribution (Figure 5C), which weakens the O─O bond and facilitates ∙OH generation, leading to enhanced peroxide‐like activity; [99] (ii) Ligand type. Replacing one N ligand with other heteroatoms (e.g., P, S, B, and O) can further tune electronic structure. For example, substituting N with the softer electron–donor P in Fe–N_4_ to form Fe–N_3_P increases electron donation to the Fe center, lowering the activation barrier for generating reactive oxygen intermediates (Figure 5D,E). As a result, the Fe–N_3_P site exhibits a specific peroxidase‐like activity nearly an order of magnitude higher than that of conventional Fe–N_4_, approaching the performance of natural HRP; [100] (iii) multisite cooperation. Dual‐atom configurations offer a distinct mode of regulation that arises from cooperation between adjacent metal centers. For example, in Zn/Mo dual‐single‐atom nanozymes, theoretical analysis suggests a stepwise mechanism: H_2_O_2_ dissociates preferentially on the Mo site owing to its stronger affinity for hydroxyl species, after which the ∙OH intermediate can migrate to the adjacent Zn site with a low energy barrier. Because Zn binds hydroxyl relatively weakly, the subsequent reaction between OH* and TMB proceeds with a smaller barrier. Such cooperation between the two metal sites helps explain the higher peroxidase‐like activity of the dual‐site nanozyme (Figure 5F,G) [101]. Besides being anchored on carbon‐based supports, single‐atom sites can also be formed on metal or metal oxide supports in the form of single‐atom alloys. In these systems, isolated hetero‐metal atoms are atomically dispersed into a catalytically active host metal, allowing the guest atom to electronically modulate the surrounding metal surface while simultaneously functioning as an active site. For example, in a Pt_1_Pd single‐atom alloy nanozyme, isolated Pt atoms are dispersed on the Pd surface and perturb the local electronic environment of adjacent Pd atoms, which results in higher peroxidase‐like activity compared with pure Pd [102].

### 2.4 Surface Functionalization

Surface ligands can modulate the catalytic activity of PMNs by regulating their affinity toward key catalytic participants, including substrates, H_2_O_2_, and reactive intermediates [103]. For substrate affinity, DNA‐capped Fe_3_O_4_ nanoparticles, as an example, display nearly 10‐fold higher peroxidase‐like efficiency toward TMB oxidation than bare Fe_3_O_4_ because the negatively charged phosphate backbone and nucleobases attract the partially protonated TMB [104]. In contrast, ABTS bears sulfonate groups and remains negatively charged, resulting in strong electrostatic repulsion with the DNA coating and consequently suppressing its oxidation. For H_2_O_2_ affinity, introducing histidine residues onto Fe_3_O_4_ creates an imidazole‐containing microenvironment that facilitates hydrogen‐bond interactions with H_2_O_2_, thereby stabilizing its binding and enhancing peroxidase‐like efficiency by up to 20‐fold compared with unmodified particles [105]. For intermediate affinity, polystyrene sulfonate ligands withdraw electron density from the surfaces of ruthenium nanozymes and weaken their ·OH adsorption, which lowers the energy barrier for substrate oxidation and markedly boosts peroxidase‐like specific activity beyond that of HRP. When used as catalytic reporters in ELISA for human alpha‐fetoprotein detection, these PMNs provided a 140‐fold improvement in detection sensitivity compared with conventional HRP‐based ELISA [106].

Before being used as catalytic reporters or labels in biosensing platforms, PMNs generally require appropriate surface functionalization to ensure colloidal stability and reliable biorecognition. For PMNs with limited aqueous dispersibility, introducing hydrophilic polymers such as polyvinylpyrrolidone (PVP) or polyethylene glycol (PEG) is often necessary to achieve stable dispersion. Once dispersibility is secured, two additional components become essential: bioreceptors (e.g., antibodies and aptamers) that provide target specificity and blocking agents that suppress nonspecific adsorption. Bioreceptors can be attached to the nanozymes through covalent or non‐covalent techniques, with each technique offering distinct advantages, and more detailed comparisons can be found in our previous reviews [31, 37]. Another important parameter is the number of bioreceptors attached per nanozyme during conjugation, which directly influences both bioassay performance and background signal. In our recent work, increasing the antibody loading from an average of 3 to 7 and then to 10 antibodies per Pt–Co PMN gradually reduced their catalytic activity, likely due to the shielding of the surface active sites [107]. However, in ELISA for cancer biomarker detection, conjugates bearing approximately 7 antibodies per PMN produced the highest signal‐to‐noise ratio, which suggests that this loading provides an optimal balance between analyte‐binding affinity and catalytic efficiency.

## 3 Synthetic Approaches

PMNs can be prepared through both chemical and physical approaches. Although physical methods such as laser ablation and mechanical exfoliation have been explored [108, 109], solution‐phase chemical synthesis remains the most widely used strategy because it offers superior control over composition, morphology, and batch‐to‐batch reproducibility. This section focuses on the solution‐phase synthetic approaches for PMNs, which can be broadly categorized into template‐free and template‐assisted methods.

### 3.1 Template‐Free Methods

One‐pot synthesis represents a versatile type of template‐free approach for PMN preparation. Direct reduction and co‐reduction are widely used to produce noble metal (e.g., Pd or Pt polyhedrons) [86, 110] and noble metal alloyed (e.g., Ni–Pt and Pt–Co nanoparticles) [88, 107] PMNs. These reactions typically involve a metal precursor, a solvent, a reductant, and a colloidal stabilizer. When conducted in polar solvents such as water or polyols, the resulting nanostructures can be readily dispersed in aqueous media, while stabilizers such as citrate or PVP provide colloidal stability and biocompatibility crucial for subsequent bioassay integration. Co‐precipitation is another commonly employed route for generating metal oxide PMNs, such as Fe_3_O_4_ [22], CeO_2_ [111], and MnO_2_ [112]. By tuning precursor ratios, pH, and ionic conditions (e.g., ionic strength and background ion species) [113], this method directly yields particles with mixed‐valence surfaces or oxygen‐vacancy‐rich domains that are critical to peroxidase‐like catalysis. However, the oxides obtained by co‐precipitation generally exhibit poor aqueous dispersibility and therefore require additional surface modification before use in bioassay applications. Hydrothermal and solvothermal reactions, in which elevated temperatures and autogenous pressures govern nucleation and crystal growth, provide access to more homogeneous metal, metal oxides, chalcogenides, and layered nanostructures with tunable crystallinity, porosity, and defect density. For example, a one‐pot hydrothermal method produces ultrasmall Ir nanoparticles (∼1.1 nm) deposited on WO_2.72_ nanorods, where the hydrophilic, defect‐rich support prevents Ir particle aggregation and yields a high *K*_cat‐specific_ [114]. Despite their simplicity and scalability, these one‐pot approaches often offer limited control over complex surface and electronic structure, which restricts the precision of active‐site engineering.

Self‐assembly represents another template‐free synthetic strategy, in which nanostructures form spontaneously through the intrinsic chemical and physical properties of the building units. Noncovalent interactions such as coordination, hydrogen bonding, π–π stacking, and hydrophobic association drive the organization of molecular precursors into higher‐order architectures. This bottom–up process resembles how natural enzymes assemble their functional domains, and nanozyme design can emulate this principle. A representative example is the sulfur–Fe–heme PMN constructed through the supramolecular assembly of hemin, Fmoc‐L‐cysteine, and Fe^2+^ coordination, which forms an Fe–N–S catalytic center with selective peroxidase‐like activity [115]. Nevertheless, self‐assembly approaches also face notable limitations [116]. They typically require multiple steps of chemical functionalization and, therefore, are difficult to scale up. In addition, the assembled products often exhibit limited control over uniformity and degree of aggregation, leading to poor batch‐to‐batch reproducibility. Moreover, the resulting hollow or porous structures frequently possess modest chemical and thermal stability. Classic examples include MOF and COF materials whose frameworks are fragile and often require an additional pyrolysis step to convert them into more robust carbonaceous structures.

### 3.2 Template‐Assisted Methods

Template‐assisted methods generally enable more precise control over surface geometry, elemental composition, and electronic structure than template‐free approaches. These strategies typically involve multiple processing steps and are often conducted in relatively small batches to maintain reproducibility, but they provide structural control that is difficult to achieve otherwise.

**FIGURE 6:**
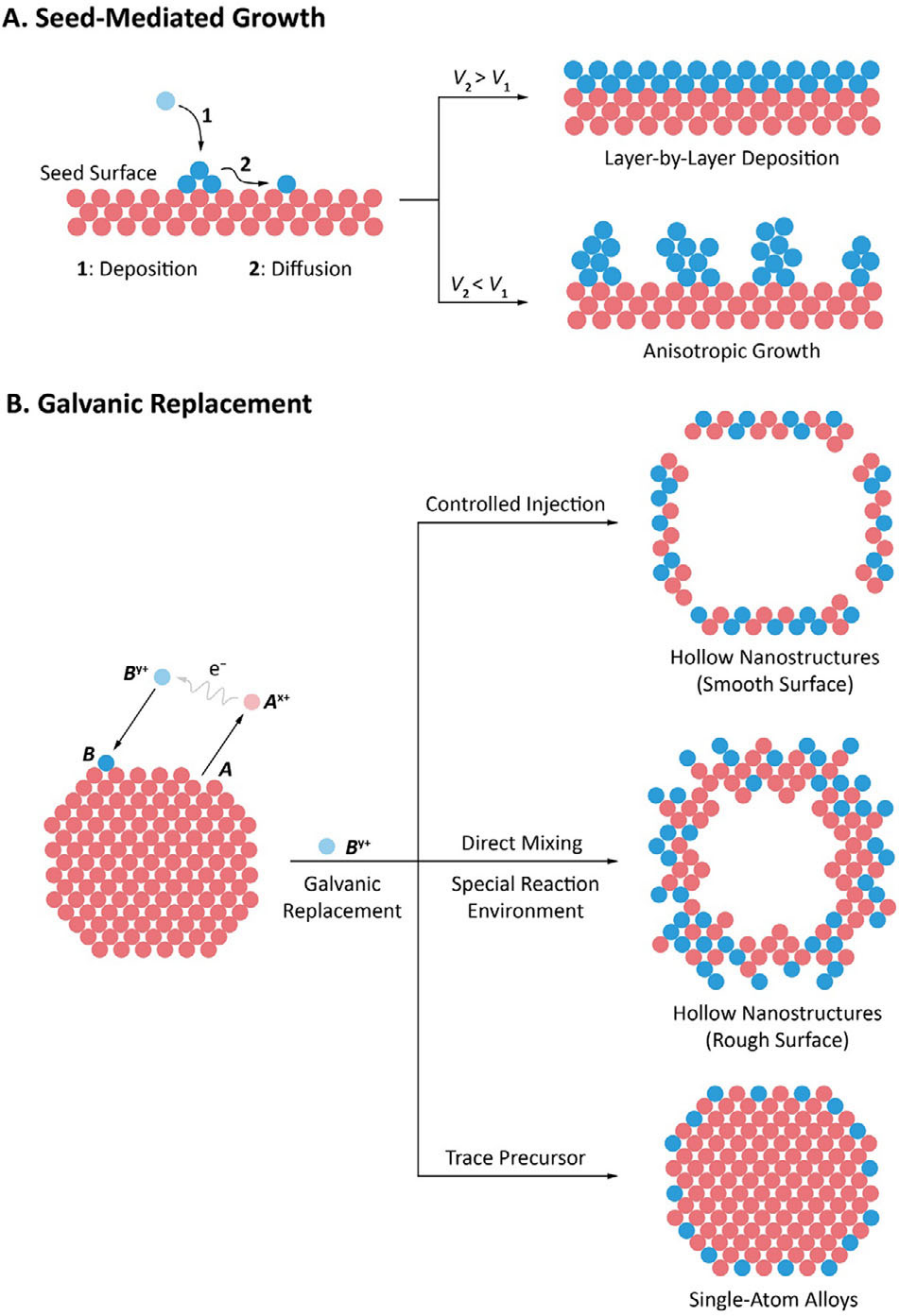
Schematics showing two representative template‐assisted synthetic approaches. (A) Seed‐mediated growth showing two pathways, where the relative rates of atom deposition and surface diffusion determine layer‐by‐layer deposition versus anisotropic growth. (B) Galvanic replacement reaction showing that variations in reaction conditions can yield diverse nanostructures, including hollow nanostructures with smooth surfaces, hollow nanostructures with rough surfaces, and single‐atom alloys.

The seed‐mediated growth method is one of the representative template‐assisted strategies (Figure 6A). In this approach, preformed seeds act as crystallographic templates onto which additional material is deposited. Growth is usually performed by continuously delivering precursors and reductant solutions by a pump, allowing the reaction kinetics at the seed surface to be finely regulated. The morphology evolution is governed by the interplay between precursor deposition/diffusion rate on the seed surface and the facet‐dependent surface energy of the seed [117]. In general, rapid precursor supply favors kinetically selective growth on specific high‐energy crystallographic facets, often leading to anisotropic or branched structures. In contrast, slow precursor feeding allows surface diffusion to dominate and promotes more uniform layer‐by‐layer deposition that is closer to thermodynamic control [37]. Deliberate manipulation of deposition kinetics, typically through injection rates, precursor‐to‐reductant ratios, the solution environment, or by tailoring the elemental composition and surface chemistry of the seed, has enabled the synthesis of PMNs with desirable structural features such as branched architectures, controlled surface strain, abundant defect sites, and multinary alloy phases. Representative examples include two types of Pd@Pt core–shell PMNs prepared by different seed‐mediated growth strategies. One strategy involves directly mixing Pd nanocubes as seeds, ascorbic acid, and a Pt precursor, which yields Pd@Pt nanodendrites [77]. Their rough, dendritic shells expose abundant high‐index facets and provide a large active surface area, resulting in strong peroxidase‐like activity and high sensitivity in ELISA for IL‐6 detection. In contrast, another strategy uses the same seeds but feeds the Pt precursor at a controlled rate using a syringe pump, producing Pd@Pt nanocubes with uniform ultrathin Pt shells [87]. These conformal shells generate well‐defined strain and ligand effects on the Pd surface, which likewise enhances peroxidase‐like activity and supports sensitive IL‐6 detection.

Galvanic replacement reaction represents another important synthetic strategy based on self‐templating (Figure 6B). In a standard synthesis, a sacrificial metal nanocrystal (metal *A*) is oxidized by ions of a second metal (metal *B*) with a higher reduction potential. During this process, atoms of metal *A* are oxidized to *A* ^x+^ and dissolve into the solution. The electrons released are then taken up by *B* ^y+^ ions, which are reduced and deposit onto the surface of the template. Such a process allows the formation of diverse alloyed nanostructures whose final morphologies depend on the shape of the sacrificial template, the precursor amount and feeding rate, and the reaction conditions [116, 118]. The resulting nanostructures typically fall into the following categories: (i) hollow alloyed nanostructures with smooth surfaces. When the precursor injection is constant and the reaction conditions are steady, the full consumption of the sacrificial template yields products that generally retain the overall morphology of the template while developing a hollow interior and a porous *A*‐*B* alloyed shell. For example, Ag–Pt hollow PMNs with a uniform alloy shell can be synthesized through galvanic replacement between Ag nanoparticles and Pt^2+^ ions, in which the K_2_PtCl_4_ precursor is injected at a controlled rate using a syringe pump; [119] (ii) Roughened or nanoflower‐like hollow nanostructures. Under special environments such as sonication, alkaline conditions, or the presence of reductants, the reaction can induce surface reconstruction and yield bumpy or nanoflower‐like shells. These roughened surfaces provide an abundance of exposed catalytic sites and are therefore favorable for peroxidase‐mimicking catalysis. For example, the VO_x_@EGaIn–PEG core–shell PMN was synthesized via galvanic replacement between liquid metal eutectic gallium–indium alloy (EGaIn) droplets and orthovanadate ions. The reaction is carried out by directly mixing the two precursors, followed by sonication, during which VO_x_ nanosheets grow outward to form a flower‐like shell that exhibits strong peroxidase‐like activity. The liquid‐metal EGaIn core imparts a unique self‐healing property, allowing the PMN to recover its structure after damage. Notably, the regenerated PMN obtained through another round of galvanic replacement maintains the original core–shell structure and preserves high peroxidase‐like activity over repeated cycles; [120] (iii) Single‐atom alloy structures. When only trace amounts of metal‐*B* precursors are introduced, the newly deposited metal atoms can remain atomically dispersed on the surface of metal *A*, forming single‐atom alloy surface configurations. For example, Pt_1_Pd single‐atom alloy PMNs were obtained through a galvanic replacement reaction between Pd nanosheets and chloroplatinic acid at a Pt:Pd molar ratio below 5%. In this configuration, isolated Pt atoms are embedded into the Pd lattice, which markedly enhances the peroxidase‐like activities of the catalyst [102].

It is worth noting that highly complex hierarchical PMNs are usually constructed by integrating multiple synthetic strategies. For example, the magnetic virus‐like nanozyme Fe–Pt_20_–Au@Pt_5_ mentioned above showed both enhanced peroxidase‐like activity and bacterial capture efficiency [71]. These particles were prepared through a sequential combination of solvothermal synthesis, colloidal reduction, seed‐mediated growth, and self‐assembly. In this design, Fe_3_O_4_ nanoparticles were first synthesized as the magnetic core by the solvothermal method. Then, 20 nm Pt nanoparticles and 5 nm Au@Pt core–shell nanoparticles were both prepared by direct chemical reduction followed by seed‐mediated growth. These nanoparticles were subsequently assembled onto the Fe_3_O_4_ core in a layer‐by‐layer manner through polyethyleneimine (PEI)‐mediated electrostatic self‐assembly, forming the virus‐like hierarchical shell.

## 4 Biomarkers for Detection of Infectious Diseases

Once PMNs are designed and synthesized, identifying appropriate biomarkers becomes a critical step for developing sensitive and clinically useful diagnostic platforms. Biomarkers relevant to infectious diseases can be broadly grouped into three major categories: (1) pathogen‐derived markers such as nucleic acids, structural proteins, or secreted antigens; (2) host immune response markers such as pathogen‐specific antibodies, cytokines, and chemokines; and (3) indirect physiological or biochemical markers associated with infection or tissue damage. Some of these biomarkers circulate at very low concentrations, especially during early infection, which can fall outside the detection range of protein enzymes, whereas others are relatively abundant in clinical samples but serve as important indicators that need to be monitored throughout treatment. In both situations, PMN‐based rapid biosensing platforms offer notable advantages over those platforms based on protein enzymes (see Section 5 for details). This section discusses the different types of biomarkers that need to be detected or monitored during the management of infectious diseases, and it also highlights key biomarkers used for the detection of representative infectious diseases (Table 1). More comprehensive information on infectious disease biomarkers can be found in previously published reviews [121, 122].

**TABLE 1:** Key biomarkers for representative infectious diseases.

Disease	Key biomarkers	Type	Typical abundance^a)^	Diagnostic relevance	Refs.
Covid‐19	N protein, S1/RBD	Antigen	pg–ng mL^−1^	Early detection	[125, 126]
	IgM, IgG, IgA	Antibody	µg–mg mL^−1^	Serology for staging and immunity	[132]
	IL‐6	Cytokine	pg–ng mL^−1^	Severity assessment	[135]
HIV	P24	Antigen	pg–ng mL^−1^	Early detection; disease monitoring	[195]
	Viral RNA	Nucleic acid	∼10^4^–10^5^ copies mL^−1^; <50 copies mL^−1^ (ART)	Treatment monitoring; perinatal transmission risk assessment	[196, 197]
	IgG, IgM	Antibody	µg–mg mL^−1^	Severity and immunity assessment	[198, 199]
Influenza A/B	Viral RNA	Nucleic acid	∼10^6^–10^7^ copies mL^−1^ (nasal swab eluate)	Complications and hospitalization assessment	[200]
	Nucleoprotein (NP)	Antigen	pg–ng per test (nasal swab)	Detection and subtype differentiation	[201]
	IgG, IgM	Antibody	µg–mg mL^−1^	Serology for staging and immunity	[133, 202]
Malaria	HRP2	Antigen	pg–ng mL^−1^	Sensitive and rapid detection	[203]
	pLDH	Antigen	pg–ng mL^−1^	Rapid detection	[203]
Tuberculosis	Mpt64 (MTB DNA)	Nucleic acid	<10^6^ copies mL^−1^ (sputum)	Early and sensitive detection	[204]
	LAM	Antigen/ metabolite	1–20 pg mL^−1^ in urine (clinical positive range)	Noninvasive detection	[131, 205]
	IFN‐γ	Cytokine	1–20 ng mL^−1^ (pleural fluid)	Diagnosis of tuberculous pleural effusion	[206]
Dengue	NS1	Antigen	Hundreds of ng to µg mL^−1^ (acute infection)	Early detection and severity indicator	[207]
	IL‐10	Cytokine	∼10–100 pg mL^−1^	Severity assessment	[207]
*Chagas* disease	Anti‐*T. cruzi* IgG	Antibody	µg–mg mL^−1^	Serologic diagnosis of chronic infection	[129, 134]

^a)^ Concentrations not explicitly labeled with a sample type refer to typical abundances in blood‐derived samples (serum or plasma). These values do not preclude detection of the same biomarkers in other clinical specimen types.

### 4.1 Pathogen‐Derived Biomarkers

Pathogen‐derived biomarkers are the primary analytical targets in infectious disease diagnostics because they directly reflect the presence and identity of a microorganism. These biomarkers include proteins, nucleic acids, and pathogen‐specific metabolites that are released, secreted, or structurally embedded in viruses, bacteria, parasites, or fungi. In addition to these classes, pathogen‐derived lipids and glycoproteins have also emerged as relevant diagnostic targets in several infectious diseases [123]. Their abundances in clinical samples often fall within the picogram (pg) to nanogram (ng) per milliliter (mL) range during the early‐stage infection and may be present only transiently or at even lower levels in chronic or low‐parasitemia infections [124]. Moreover, required sample pretreatment steps‐such as dilution, matrix removal, or nucleic acid extraction‐further reduce the effective concentration of analytes available for detection, underscoring the need for ultrasensitive analytical platforms capable of capturing and amplifying scarce pathogen‐derived targets.

Antigenic proteins represent the most widely used class of biomarkers in rapid diagnostic tests. Viral structural proteins are among the most abundantly expressed components during early infection and provide strong specificity for viral identification. For example, COVID‐19 diagnostics commonly target the spike (S1/RBD) and nucleocapsid (N) proteins, which are the two principal SARS‐CoV‐2 antigens used in serological and antigen tests. The N protein is highly abundant in early infection and offers high sensitivity for early diagnosis, whereas S1 and its receptor‐binding domain (RBD) provide strong specificity and are widely applied in serological surveillance [125]. Despite their high expression within infected cells, the concentrations of these antigens released into clinical specimens remain low. Quantitative measurements show that N‐protein in plasma typically ranges from 10 to 1000 pg mL^−1^ during early infection [126]. Similarly, bacterial pathogens express conserved surface structures for binding, such as lipopolysaccharides (LPS), peptidoglycan, and unique surface proteins like protein A in *Staphylococcus aureus* (*S. aureus*, a major Gram‐positive pathogen frequently associated with skin, soft‐tissue, and systemic infections) [127]. A key distinction between Gram‐positive and Gram‐negative bacteria lies in their cell envelope structure, which influences antigen accessibility during detection [128]. Gram‐positive bacteria contain a thick peptidoglycan layer that leaves many surface antigens readily accessible for detection, whereas Gram‐negative bacteria possess an LPS‐rich outer membrane that can partially mask antigenic sites and reduce binding accessibility. As a result, detection strategies for Gram‐negative bacteria often target the exposed components, such as LPS or specific outer membrane proteins. Parasitic infections also produce distinctive antigenic targets. However, many parasite‐derived antigens are not present at detectable levels in clinical samples and are therefore used in recombinant form for serological assays. For example, in *Chagas* disease, the *Trypanosoma cruzi* Tc24 protein is a conserved and diagnostically useful antigen that is commonly used to capture and detect anti‐*T. cruzi* IgG in serological assays. This parasitic disease remains highly endemic in Latin America [129].

Beyond surface proteins, nucleic acid biomarkers provide highly specific signatures for pathogen detection. Viral RNA sequences, including conserved genomic regions and subtype‐defining fragments, are central to early diagnosis of diseases such as COVID‐19 and HIV. Bacterial ribosomal RNA and species‐specific DNA sequences also serve as powerful molecular identifiers. Although nucleic‐acid detection traditionally requires enzymatic amplification such as PCR or loop‐mediated isothermal amplification, emerging nanozyme biosensors have integrated target‐responsive functional DNA structures with nanozyme‐mediated signal amplification to achieve enzyme‐free or low‐enzyme molecular diagnostics [65, 130].

Pathogens also produce metabolites that function as clinically relevant biomarkers. For example, in tuberculosis, lipoarabinomannan (LAM) is a major glycolipid released by *Mycobacterium tuberculosis* and is detectable in urine, making it one of the most promising noninvasive tuberculosis biomarkers [131].

### 4.2 Host Immune Response Biomarkers

Host immune responses offer another key group of biomarkers for infectious disease diagnostics. They help distinguish stages of infection, assess disease severity, and complement pathogen‐derived antigen detection. These biomarkers primarily include antibodies, cytokines, and acute‐phase proteins, all of which appear in peripheral biofluids at clinically relevant concentrations and are compatible with PMN‐based immunoassays.

First, antibodies such as IgM, IgG, and IgA are widely used serological indicators. IgM typically emerges during the early phase of infection and serves as a marker of recent exposure, while IgG reflects later‐stage or past infection and is frequently used for immunity assessment. In respiratory infections, including COVID‐19 and influenza A/B, the temporal pattern of antibody appearance (e.g., IgA and IgM rising earlier than IgG) provides diagnostic value for determining infection stage [132, 133]. These immunoglobulins are present in serum at µg to mg mL^−1^ levels, and multiplexed detection platforms that measure several isotypes simultaneously can help delineate the stage of infection while also improving diagnostic sensitivity and specificity. In parasitic diseases such as *Chagas* disease, host antibody responses remain the cornerstone of diagnosis, as direct pathogen‐derived biomarkers are often below detectable levels in chronic infection. However, variability in *Trypanosoma cruzi* antigenic epitopes across different genotypes can lead to discordant serological results, underscoring the need for antigen panels or conserved epitope–based assays capable of improving sensitivity and cross‐strain performance [134].

Second, cytokines and chemokines act as markers of inflammation and disease severity. For example, interleukin‐6 (IL‐6) is elevated in severe viral infections such as COVID‐19 and is a strong prognostic indicator [135]. Tumor necrosis factor‐α (TNF‐α) and interferon‐γ (IFN‐γ) reflect the activation of antiviral or antibacterial immune pathways and are associated with severe inflammatory responses and sepsis progression [136]. These cytokines circulate in serum or plasma at low picogram‐to‐nanogram levels [137].

Third, acute‐phase proteins provide additional clinically actionable markers. For instance, C‐reactive protein increases rapidly during bacterial pneumonia, tuberculosis, and systemic inflammation, and its concentrations are generally higher in bacterial than in viral infections, making it a useful triage marker in primary care settings. Procalcitonin is another widely adopted biomarker that also helps differentiate bacterial from viral infections and informs antibiotic treatment decisions [138].

### 4.3 Indirect Markers

Indirect biomarkers reflect physiological disturbances caused by infection rather than the presence of the pathogen itself. Although they lack pathogen specificity, these biomarkers provide clinically valuable information for evaluating disease severity, guiding treatment decisions, and supporting early triage, particularly in complex clinical settings where pathogen‐derived biomarkers may be below detectable levels. Among the most widely used indirect biomarkers are enzymes released during tissue injury, such as alanine aminotransferase (ALT) and aspartate aminotransferase (AST) [139, 140]. Elevated ALT and AST levels indicate hepatocellular damage and are frequently observed in viral hepatitis, HIV coinfections, and other systemic infections that affect liver function. These enzymes provide actionable information for assessing organ involvement and monitoring disease progression, complementing pathogen‐specific assays. Another important indirect marker is lactate, a metabolic byproduct that accumulates during anaerobic glycolysis under conditions of tissue hypoxia or severe systemic inflammation [141, 142]. Elevated lactate levels are strongly associated with sepsis and serve as a prognostic indicator for patient outcomes. Because lactate concentration correlates with severity rather than the identity of an infection, rapid lactate detection can be crucial for early intervention in septic patients.

## 5 Biosensing Platforms for Rapid Detection

PMN‐based biosensing platforms have shown superior performance in detecting infectious diseases compared with platforms that rely on natural peroxidases. Particularly, PMN‐based sensing platforms can be engineered to show higher sensitivity within concentration ranges that enzymes cannot reliably cover, and they can provide a broader linear dynamic range. These features reduce the sample volume required for each measurement and facilitate minimally invasive sampling. As a result, even abundant biomarkers that require daily or frequent monitoring for surveillance, triage, treatment decisions, or prognostic assessment can be measured with minimal sample input, which improves patient comfort. In addition, PMNs are reliable in complex clinical matrices. Some biomarkers are associated with inflammation‐triggered conditions in which proteases are overexpressed, and in these cases, protein enzymes may be degraded in clinical samples and generate false‐negative signals. In contrast, PMN‐based platforms can maintain catalytic activity and ensure analytical reliability. Moreover, PMNs are compatible with a wide range of environmental conditions, which enables on‐site detection in regions with extreme climates, such as cold near‐polar regions or hot and humid near‐equatorial regions. In this section, we review PMN‐enabled biosensing platforms that align with practical needs and are consistent with the WHO ASSURED criteria, which emphasize affordability, sensitivity, specificity, user‐friendliness, rapidity, equipment‐free operation, and deliverability (Table 2) [143].

**TABLE 2:** PMN‐based sensing platforms for rapid detection of infectious diseases.

Pathogen/Disease	Nanozymes	Analyte	Sensing platform	Limit of detection	Refs.
SARS‐CoV‐2	FeS_2_	Nucleic acid	Colorimetric lateral flow assay	200 copies mL^−1^	[53]
SARS‐CoV‐2	Co‐Fe@hemin	Antigen	Chemiluminescent lateral flow assay	0.1 ng mL^−1^	[33]
SARS‐CoV‐2	FeAu@AuIr‐MPBA	Antigen	Multiplexed lateral flow assay	1.2 pg mL^−1^	[145]
SARS‐CoV‐2	MnO_2_QDs@Lip	Antigen	Solution‐phase colorimetric assay	65 pg mL^−1^	[153]
HPV	FeS_2_	Nucleic acid	Colorimetric lateral flow assay	500 copies mL^−1^	[53]
HIV	Pt/Ti_3_C_2_T_x_	Nucleic acid	Colorimetric lateral flow assay	0.1 nM	[84]
Influenza B	MIL‐101(CoFe)	Antigen	Colorimetric/Raman nasal swab sensor	1.3 ng mL^−1^ (Raman)	[155]
H1N1	Nanozyme‐based test strip	Antigen	Automated microfluidic device	50 pg mL^−1^	[152]
Zika virus	Pt@Au	Antigen	Colorimetric vial immunosensor	1 pg mL^−1^	[208]
Melioidosis	MXene@PtCu	Nucleic acid	Colorimetric lateral flow assay	1.26 fM	[85]
*K. pneumonia*	Hollow Au@Au@Ag/Pt	Whole‐cell bacteria	Multi‐mode lateral flow assay	10^3^ CFU mL^−1^ (Catalytic)	[209]
*S. aureus*	Fe_3_O_4_@MOF@PtPd	Whole‐cell bacteria	Colorimetric lateral flow assay	2 CFU mL^−1^	[210]
*S. aureus*	Pd@Pt	Protein A	Paper‐based ELISA	9.56 ng mL^−1^	[146]
*S. aureus*	Au NPs/2D‐MOFs	Whole‐cell bacteria	Electrochemical sensor	6 CFU mL^−1^	[148]
*S. aureus*	MoO_3_/MIL‐125‐NH_2_	Whole‐cell bacteria	Electrochemical sensor	16 CFU mL^−1^	[150]
*S. mutans*	Fe_3_O_4_/Sm3 bioconjugate	Whole‐cell bacteria	Solution‐phase colorimetric assay	12 CFU mL^−1^	[211]
*P. aeruginosa*	Au NP	Whole‐cell bacteria	Electrochemical sensor	60 CFU mL^−1^	[149]
*P. aeruginosa*	FeAu@AuIr‐MPBA	Whole‐cell bacteria	Multiplexed lateral flow assay	17 cells mL^−1^	[145]
*S. pneumoniae*	FeAu@AuIr‐MPBA	Whole‐cell bacteria	Multiplexed lateral flow assay	21 cells mL^−1^	[145]
*Salmonella*	Au@PtPd	Whole‐cell bacteria	Finger‐actuated microfluidic device	45 CFU mL^−1^	[151]
*H. pylori*	Fe‐Pt_20_‐Au@Pt_5_	Whole‐cell bacteria	Multiplexed lateral flow assay	4 CFU mL^−1^	[71]
*S. typhi*	Fe‐Pt_20_‐Au@Pt_5_	Whole‐cell bacteria	Multiplexed lateral flow assay	5 CFU mL^−1^	[71]
*E.coli* O157:H7	Fe‐Pt_20_‐Au@Pt_5_	Whole‐cell bacteria	Multiplexed lateral flow assay	9 CFU mL^−1^	[71]
Antibiotic‐resistant bacteria	Au‐Fe_3_O_4_	Nucleic acid	Colorimetric CRISPR‐Cas12a assay	<0.1 CFU µL^−1^	[154]

### 5.1 Paper‐Based Assays

Among paper‐based formats, the lateral flow assay (LFA) is one of the most widely used POC diagnostic techniques owing to its rapid turnaround, simple operation, and low cost. In a conventional sandwich‐type LFA, gold nanoparticles (typically around 40 nm) are used as labels, and the signal readout originates from their plasmonic properties, which fundamentally limit sensitivity. In contrast, in LFAs powered by PMNs, the labels are replaced by catalytic nanomaterials (i.e., PMNs), and the signal is generated through oxidation of the added substrates. This will require an additional substrate treatment process after the normal LFA procedure. After the chromatographic migration process, a substrate containing hydrogen peroxide and a reporter substrate is applied to the test/control line regions and allowed to react for several minutes at room temperature. The signal intensity originated from the amount of the oxidized reporter substrates generated during the treatment process, and this amount is determined by the intrinsic peroxidase‐like activity of the PMN labels. In practice, this signal can surpass the plasmonic response of conventional gold nanoparticles by several orders of magnitude. For example, Pt‐based nanoparticles used as catalytic labels could have an equivalent molar extinction coefficient of 1.8 × 10^13^ m^−1^ cm^−1^ after 5 min of TMB oxidation, which is approximately three orders of magnitude higher than the molar extinction coefficient of ∼40 nm Au nanoparticles [144].

**FIGURE 7:**
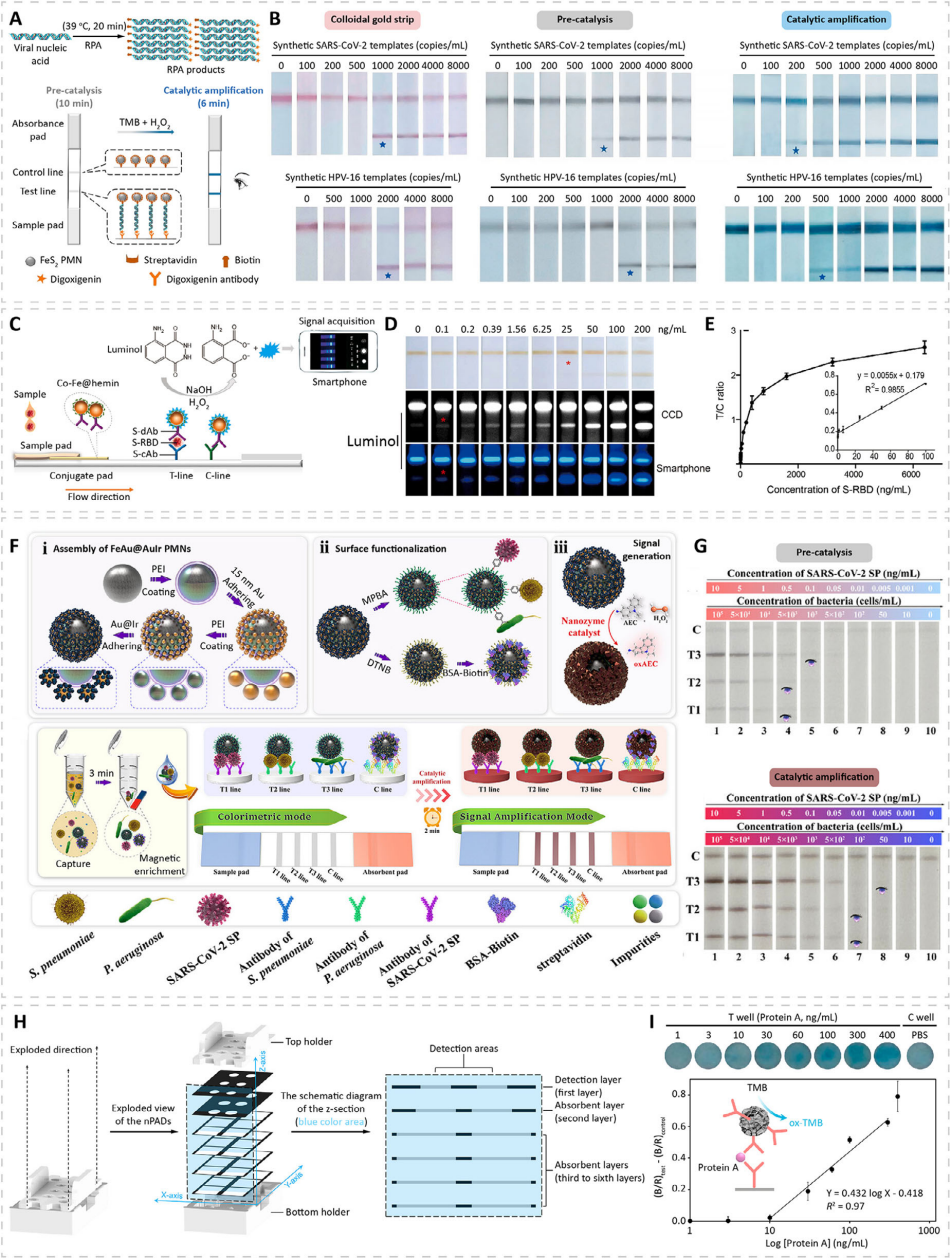
Examples of PMN‐based rapid sensing using paper‐based assays. (A,B) Colorimetric lateral flow assays (LFAs) using PMNs to catalyze chromogenic substrates. (A) Schematics showing a colorimetric lateral flow assay using FeS_2_ PMNs combined with recombinase polymerase amplification for nucleic acid detection of SARS‐CoV‐2 and human papilloma virus (HPV). (B) Photographs of test strips comparing conventional colloidal gold‐based LFAs, FeS_2_ PMN‐based LFAs before catalytic amplification, and the same strips after TMB–H_2_O_2_ development. Reproduced with permission [53]. Copyright 2022, Elsevier. (C–E) Chemiluminescent LFAs using PMNs to catalyze luminol substrates. (C) Schematics showing a chemiluminescent LFA based on Co–Fe@hemin PMNs for detecting the SARS‐CoV‐2 S‐RBD protein. (D) Photographs of test strips before and after the addition of luminol substrate, and (E) the corresponding calibration curve. Reproduced with permission [33]. Copyright 2020, Elsevier. (F,G) PMN‐based multiplexed LFAs. (F) Schematics showing the preparation of MPBA‐modified FeAu@AuIr PMNs and their use as catalytic labels for multiplexed LFA detection of respiratory pathogens. (G) Photographs of test strips showing simultaneous detection of *S. pneumoniae*, *P. aeruginosa*, and SARS‐CoV‐2 spike protein before (upper) and after (lower) AEC–H_2_O_2_ development. Reproduced under CC BY license. (H,I) Other paper‐based devices using PMNs as catalytic reporters. (H) Schematic diagram of a Pd@Pt nanozyme‐immobilized paper‐based analytical device (nPAD) showing the exploded structure and z‐section side view of the wax‐printed paper layers assembled within a 3D‐printed sample holder. (I) Detection of protein A from *S. aureus* using the nPAD, with representative photographs of the test and control wells (upper) and the corresponding calibration curve (lower). Reproduced with permission [146]. Copyright 2022, Elsevier.

The chromogenic substrates used in PMN‐based LFAs are typically selected to generate insoluble colored products upon catalytic oxidation, which provides strong visual contrast against the nitrocellulose membrane. Common chromogenic substrates include TMB, which yields a blue oxidized product, and AEC, which yields a red product. In an example, FeS_2_ PMNs were used as catalytic labels in LFA for nucleic acid detection of SARS‐CoV‐2 (an RNA virus) and human papilloma virus (HPV, a DNA virus) [53]. By integrating recombinase polymerase amplification (RPA) with FeS_2_ probes, the assay produced amplified signals after substrate development and achieved a LOD of 200 copies mL^−1^ for SARS‐CoV‐2 and 500 copies mL^−1^ for HPV within 36 min. In comparison, the corresponding colloidal gold‐based LFA detected only 1000 and 2000 copies mL^−1^, respectively (Figure 7A,B). Besides chromogenic substrates, chemiluminescent substrates can also be used in PMN‐based LFAs. For example, Co‐Fe@hemin PMN‐based LFA was developed for detecting SARS‐CoV‐2 spike antigen (S‐RBD antigen) [33]. The PMNs catalyzed the oxidation of luminol in alkaline solution, which generated the chemiluminescent readout. The assay reached a detection limit of 0.1 ng mL ^−1^ with a linear range of 0.2–100 ng mL^−1^ (Figure 7C–E). Notably, luminol oxidation requires alkaline conditions, whereas most PMNs reported and HRP function optimally in acidic media, thus PMNs must be rationally designed to maintain activity in alkaline environments. In this case, the Co‐Fe@hemin nanozyme remained active across pH range from 9 to 14 and showed excellent temperature tolerance, supporting reliable chemiluminescent readout in the LFA format. The test can be performed using a portable device or a smartphone with camera, which aligns with the ASSURED criteria.

In some cases, multiplexed detection is needed, and the LFA format can accommodate multiple test lines for identifying different targets. For example, bacterial and viral respiratory infections often present similar symptoms but require distinct treatment strategies. In this context, FeAu@AuIr PMNs with multi‐tentacle shells were functionalized with 4‐mercaptophenylboronic acid (MPBA) to enable broad‐spectrum binding to peptidoglycan in bacterial cell walls and to viral envelop glycoproteins [145]. The spiked or clinical samples in this study contained one or multiple analytes, including *Streptococcus pneumoniae*, *Pseudomonas aeruginosa*, and SARS‐CoV‐2 virus, which are all directly related to respiratory infections. After mixing them with the MPBA‐modified PMNs, these pathogens were captured within 5 min and subsequently subjected to immunochromatographic separation on a test strip with three pathogen‐specific test lines. Under substrate treatment in the AEC–H_2_O_2_ system, the assay achieved detection sensitivities of 21 cells mL^−1^ for *S. pneumoniae*, 17 cells mL^−1^ for *P. aeruginosa*, and 1.2 pg mL^−1^ for SARS‐CoV‐2. These sensitivities represent ∼16–73 times improvement compared to pre‐catalysis signals and 238–416 times enhancement over conventional gold nanoparticle‐based colorimetric LFA (Figure 7F,G).

Except LFA, other paper‐based devices have also been developed using PMNs. A representative example is a nanozyme‐immobilized paper‐based analytical device (nPAD), in which a paper‐based ELISA format is integrated with Pd@Pt PMNs as the catalytic reporter for detecting Protein A from *S. aureus* (Figure 7H,I) [146]. The device consists of a 3D‐printed top holder, a wax‐patterned paper detection layer, five underlying wax‐patterned absorbent layers, and a 3D‐printed bottom holder assembled in a vertical flow configuration (Figure 7H). Wax patterning defines hydrophilic wells on the detection layer that serves as the immunoassay testing zones, while the absorbent layers wick excess liquid away from these wells. The wells are chemically modified with crosslinkers for covalent coupling of ant‐Protein A antibodies to form detection wells and then blocked with bovine serum albumin (BSA) to minimize nonspecific binding. During the assay, only a few microliters of sample are added to the test wells. If Protein A is present, it binds to the immobilized antibody and subsequently interacts with the anti‐Protein A‐functionalized Pd@Pt PMNs. Upon addition of TMB substrate, the PMNs catalyze its oxidation to produce a blue color that can be read by eye or with a smartphone. The shortened diffusion distance and reduced reagent consumption allow the assay to be completed in about 30 min with a much smaller sample volume than conventional plate‐based ELISA, achieving a detection limit of 9.56 ng mL^−1^ and supporting POC detection.

### 5.2 Electrochemical Sensors

Electrochemical sensors provide a sensitive and rapid platform for infectious‐disease diagnostics and are well‐suited for POC use. Many systems have already been miniaturized into low‐power, chip‐based formats that interface with handheld readers or smartphones. In a typical configuration, electrochemical biosensors place a molecular recognition element onto an electrode that serves as the signal transducing surface. When target analytes interact with the recognition components immobilized on the electrode surface, they induce local redox or interfacial changes that lead to measurable variations in current, potential, conductance, or capacitance. These electrical responses reflect the presence and concentration of the analyte and allow both qualitative identification and quantitative assessment [147].

**FIGURE 8:**
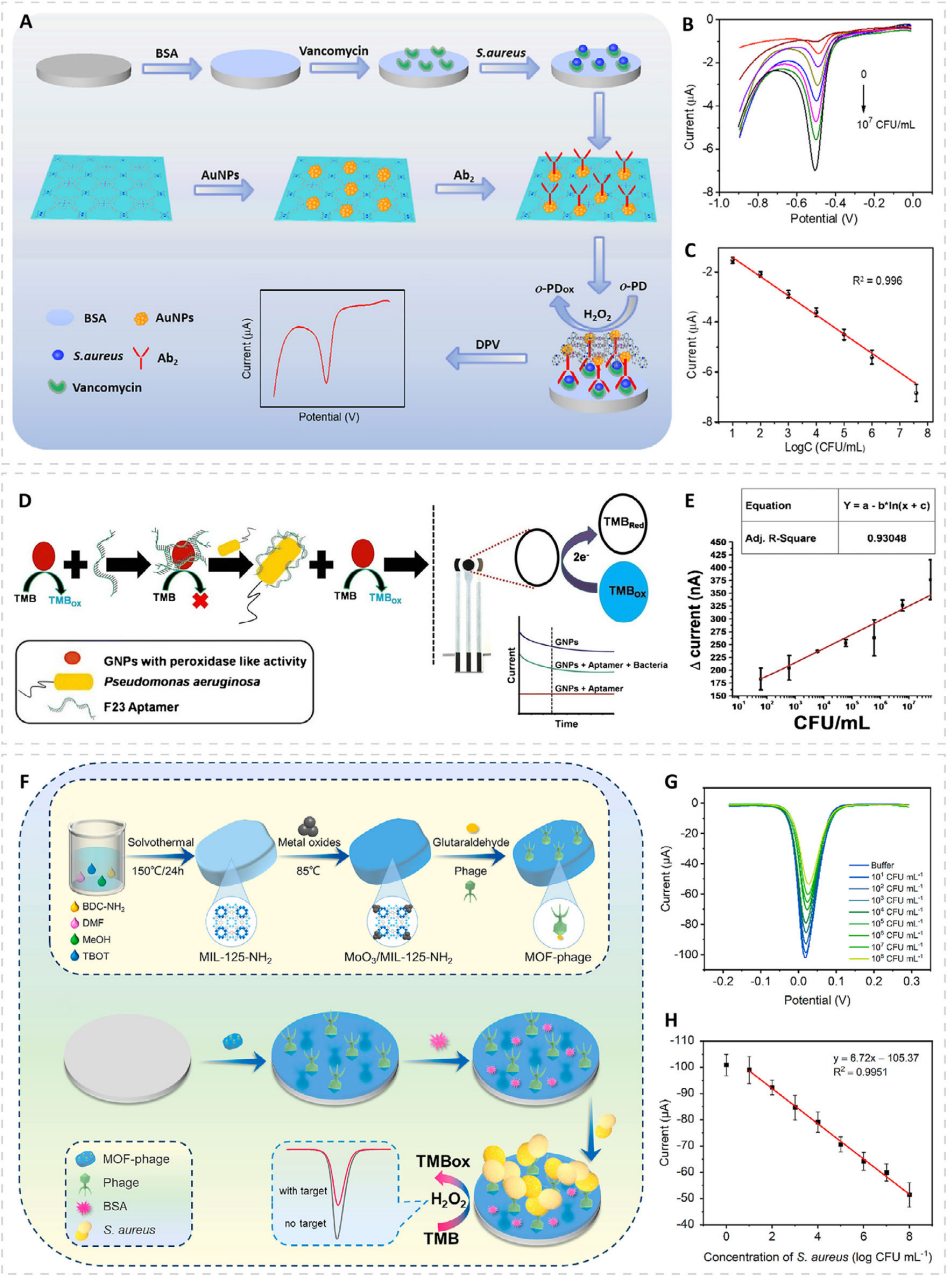
Examples of PMN‐based electrochemical sensors. (A–C) Sandwich‐type electrochemical immunoassays. (A) Schematic illustrating a sandwich‐type electrochemical immunoassay for detecting *S. aureus* using gold‐nanoparticle‐embedded 2D MOF PMNs. (B) Differential pulse voltammetry (DPV) reduction peaks obtained at different *S. aureus* concentrations, and (C) the corresponding calibration curve. Reproduced with permission [148]. Copyright 2021, American Chemical Society. (D,E) “Turning‐on” strategies. (D) Schematic illustrating an electrochemical assay for *P. aeruginosa* in which the presence of the bacteria removes the aptamer from the gold nanoparticles, thereby restoring their peroxidase‐like activity. (E) Calibration curve of the assay. Reproduced with permission [149]. Copyright 2019, Springer Nature Limited. (F–H) “Turning‐off” strategies. (F) Schematics illustrating an electrochemical assay for *S. aureus* based on MoO_3_/MIL‐125‐NH_2_ PMNs, where adsorption of *S. aureus* on the PMN surface blocks peroxidase‐like activity. (G) DPV responses at varying *S. aureus* concentrations, and (H) the corresponding calibration curve. Reproduced with permission [150]. Copyright 2024, Elsevier.

Among electrochemical biosensing formats, the use of PMNs most commonly appears in sandwich‐type electrochemical immunoassays, where PMNs replace HRP to generate electroactive products (e.g., oxidized TMB or OPD) for voltammetric readout. For example, an electrochemical detector was constructed from a 2D MOF nanozyme decorated with in situ‐grown gold nanoparticles (Figure 8A–C) [148]. This hybrid nanozyme was functionalized with anti‐*S. aureus* antibodies and served as the secondary recognition element. Meanwhile, vancomycin was immobilized on the electrode surface to capture *S. aureus* and form the primary recognition interface. Once the sandwich‐type immunoassay was assembled through antibody recognition, the MOF nanozyme catalyzed the oxidation of OPD in the presence of H_2_O_2_ to produce 2,2‐diaminoazobenzene (OPDox), an electroactive species that is reduced back to OPD at the electrode, yielding a distinct reduction peak in differential pulse voltammetry (DPV). This approach enabled ultrasensitive detection of *S. aureus* with a detection limit of 6 CFU mL^−1^ and high selectivity over other bacteria.

“Turning on” strategies can also be implemented in electrochemical sensing by utilizing target‐induced recovery of PMN activity. A representative example is an aptamer‐gold nanoparticle system for detecting *P. aeruginosa* (Figure 8D,E) [149]. Gold nanoparticles possess peroxidase‐like activity, but adsorption of *P. aeruginosa*‐specific aptamer (F23) onto their surface effectively suppresses this activity. In the presence of the cognate bacterial target, the aptamer preferentially binds to *P. aeruginosa* and leaves the nanoparticle surface, thereby restoring the activity of the gold nanoparticles. The activated nanozyme then oxidizes TMB to its electroactive form, enabling amperometric detection on a screen‐printed carbon electrode. This target‐triggered catalytic recovery allows rapid and sensitive detection of *P. aeruginosa* with a detection limit of ∼60 CFU mL^−1^ within 10 min.

In addition to “turning on” strategies, PMN‐based electrochemical sensors can also operate through “turning off” mechanisms, in which the catalytic activity of PMNs is deliberately suppressed upon target binding. For example, MoO_3_/MIL‐125‐NH_2_ composites show outstanding peroxidase‐like activity toward the TMB–H_2_O_2_ system and generate a clear DPV reduction peak when immobilized on the electrode [150]. After the nanozyme is conjugated with *S. aureus*‐specific bacteriophages, the subsequent binding of *S. aureus* cells physically blocks the nanozyme surface and suppresses its catalytic activity (Figure 8F–H). The resulting decrease in the DPV signal is concentration‐dependent, showing a linear calibration range of 10^1^–10^8^ CFU mL^−1^ and a detection limit of 16 CFU mL^−1^.

### 5.3 Microfluidic Devices

**FIGURE 9:**
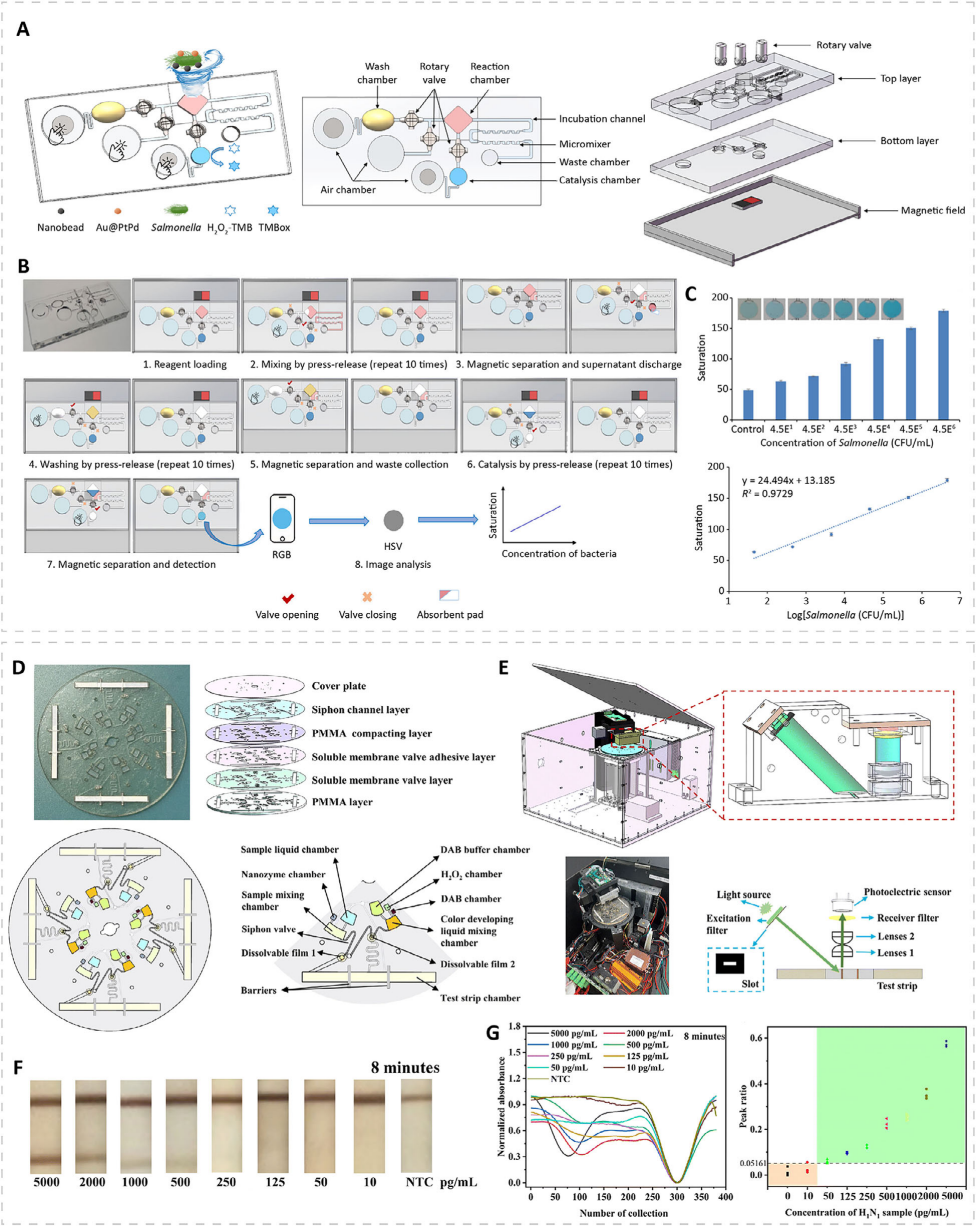
Examples of PMN‐based microfluidic devices. (A–C) Finger‐actuated microfluidic device. (A) Principle and structural layout of a finger‐actuated microfluidic device using Au@PtPd PMNs for detecting *Salmonella*. (B) Stepwise operating procedure of the device. (C) Colorimetric intensities measured at different *Salmonella* concentrations, and the corresponding calibration curve. Reproduced with permission [151]. Copyright 2024, Elsevier. (D–G) Automated microfluidic device. (D) Photograph, front view, and exploded view of a microfluidic chip used in an automated platform designed to process PMN‐based test strips for detecting H1N1 viral antigens. (E) Schematics and a photograph of the centrifugal analyzing platform equipped with an optoelectronic scanning analyzer module. (F) Photographs of the test strips obtained at the eighth minute from nine H1N1 virus samples with varying concentrations. (G) Normalized absorbance curves, corresponding peak ratios, and threshold setting used for signal discrimination. Reproduced with permission [152]. Copyright 2025, The Royal Society of Chemistry.

At an advanced level of integration, PMNs have emerged as powerful signal transducers in microfluidic devices. These devices provide an all‐in‐one platform that can be rationally designed to streamline every step of the analytic workflow, including specimen collection, sample pretreatment, reagent manipulation, bioreaction, and signal readout. Microfluidic technologies are inherently capable of accommodating multiple analytes and supporting diverse detection modes such as colorimetric, electrochemical, and fluorescence measurements. When combined with high catalytic efficiency and versatile signal outputs of PMNs, these microfluidic systems offer rapid, portable, and user‐friendly solutions that are well‐suited for the detection of infectious diseases. For example, a finger‐actuated microfluidic chip has been developed using Au@PtPd PMNs for the detection of *Salmonella* (Figure 9A–C) [151]. In this device, bacterial samples, polyclonal antibody‐modified magnetic nanobeads, and monoclonal antibody‐modified Au@PtPd PMNs are introduced into the chip and rapidly mixed through a combination of passive convergence‐and‐divergence micromixing and active extrusion‐and‐suction micromixing generated through repeated finger press‐release actions. These mixing steps promote efficient formation of nanobead‐bacteria‐PMN conjugates, which are then magnetically separated on‐chip and washed with the assistance of rotary microvalves. After removal of the unbounded components, the captured conjugates are exposed to a TMB–H_2_O_2_ substrate to yield a colorimetric signal whose intensity reflects the bacterial concentration. This chip integrates all steps from target recognition to signal generation into a single manually operated device, and the final colorimetric output can be recorded and quantified using a smartphone. The entire workflow is completed in 25 min, enabling sensitive detection of *Salmonella* with a detection limit of 45 CFU mL^−1^ without the need for external instrumentation.

Microfluidic devices can be integrated with automation accessories to further reduce user intervention and shorten assay time, which is important for high‐throughput POC screening or detection. For example, a centrifugal microfluidic chip can simultaneously load multiple PMN‐based LFA strips, and it uses motor‐driven rotation, dissolvable‐film valves, and automated fluid routing to sequentially deliver the sample and chromogenic substrate solution without manual pipetting (Figure 9D–G) [152]. The system also incorporates an optoelectronic scanning module that automatically acquires and analyzes the test and control line signals. With this fully automated workflow, H1N1 antigen detection requires only 1 min of manual handling time and is completed within 16 min, achieving a detection limit of 50 pg mL^−1^ and offering a 2.5‐fold improvement in sensitivity compared with naked‐eye readout.

### 5.4 Other Colorimetric Assays

In addition to paper‐based assays, electrochemical sensors, and microfluidic devices, PMNs can also be integrated into a variety of solution‐phase colorimetric assays. These systems typically rely on specially engineered nanostructures or assay procedures that first identify the target analyte in the specimen and subsequently trigger the enrichment or release of PMNs, which then generate a colorimetric signal whose intensity aligns with the analyte concentration.

**FIGURE 10:**
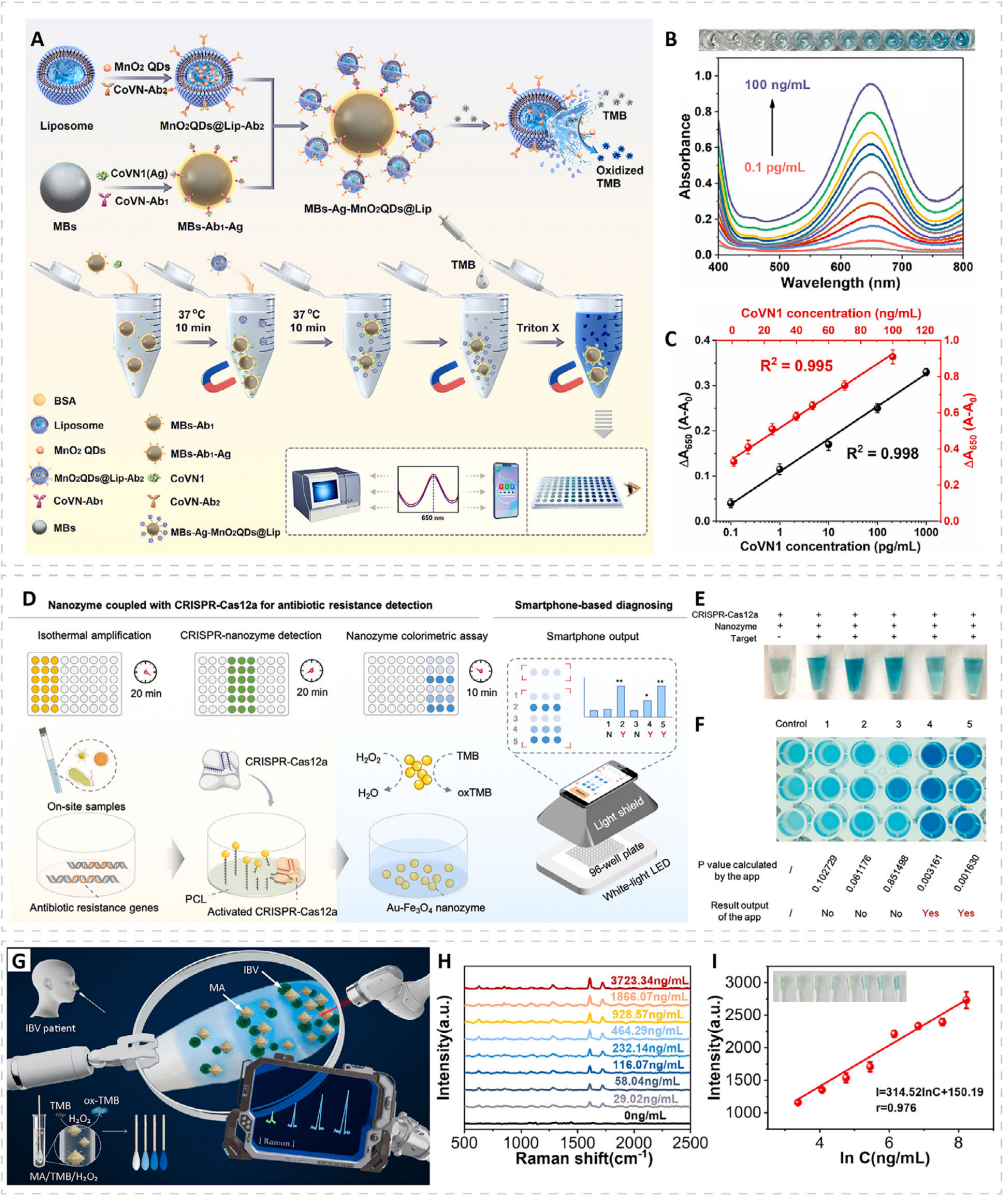
Examples of other PMN‐based colorimetric rapid detection assays. (A–C) Solution‐phase colorimetric assay based on a one‐target‐to‐multiple‐nanozyme amplification mechanism. (A) Schematic illustration of a colorimetric immunoassay using liposome‐encapsulated MnO_2_ quantum‐dot PMNs (MnO_2_QDs@Lip) for detecting the SARS‐CoV‐2 nucleocapsid antigen (CoVN1). (B) UV–Vis spectra and corresponding photographs at different CoVN1 concentrations, and (C) the calibration curve based on absorbance at 650 nm. Reproduced with permission [153]. Copyright 2024, Elsevier. (D–F) Solution‐phase colorimetric assay based on a CRISPR‐Cas‐PMN system. (D) Schematic illustration of a CRISPR‐Cas system‐assisted colorimetric assay using Au–Fe_3_O_4_ PMNs for detecting antibiotic‐resistant genes. (E) Photographs demonstrating the feasibility of the assay. (F) Representative analysis outputs from the smartphone application. Reproduced with permission [154]. Copyright 2023, Elsevier. (G–I) Direct colorimetric detection on clinical nasopharyngeal swabs. (G) Schematic illustration of a nasopharyngeal swab biosensor based on MIL‐101(CoFe) PMNs for colorimetric/SERS detection of influenza B virus (IBV). (H) Raman spectra of pharyngeal swabs containing different IBV concentrations after treatment with the sensor solution (MA) and subsequent TMB–H_2_O_2_ development, and (I) the corresponding calibration curve. Reproduced with permission [155]. Copyright 2025, Elsevier.

A representative example employs liposome‐encapsulated MnO_2_ quantum‐dot PMNs (MnO_2_QDs@Lip) to achieve highly amplified colorimetric detection of SARS‐CoV‐2 antigen (Figure 10A–C) [153]. In this system, ultrasmall MnO_2_ QDs (3–5 nm) with strong peroxidase‐like activity are packed inside lipid vesicles, enabling each vesicle to carry a large amount of quantum dot units. During detection, SARS‐CoV‐2 nucleocapsid antigens in the sample are first captured and magnetically enriched by primary antibody‐modified magnetic beads. The resulting bead‐antigen complexes then bind to secondary antibody‐functionalized MnO_2_QDs@Lip to form an immunocomplex. Upon magnetic separation, Triton X‐100 is introduced to rupture the liposomal membrane. As a result, MnO_2_ QDs are released into the solution and rapidly catalyze the TMB–H_2_O_2_ reaction, generating a colorimetric signal correlating with antigen concentration. Taking the advantages of immune‐magnetic separation and the one‐target‐to‐multiple‐nanozyme amplification mechanism, this assay achieves a detection limit of ∼65 fg mL^−1^ with a wide linear range spanning 0.1 pg mL^−1^ to 100 ng mL^−1^. The total assay can be completed within ∼20 min, and its performance in clinical samples have also been demonstrated.

Recently, the CRISPR‐Cas system has emerged as a powerful molecular recognition module that can be integrated into PMN‐based colorimetric assays. For example, Au–Fe_3_O_4_ PMNs combined with the Cas12a mechanism have been used for genotypic identification of antibiotic‐resistant bacteria, which represent an increasingly severe global health concern (Figure 10D–F) [154]. In this assay, Au–Fe_3_O_4_ PMNs are first immobilized on a polycaprolactone nanofiber membrane through biotin–avidin interactions using ssDNA as linkers. These DNA strands also function as trans‐cleavage substrates for Cas12a. Once the target resistance gene (e.g., kanamycin‐, ampicillin‐, or chloramphenicol‐ resistance genes) is amplified and recognized by the Cas12a‐crRNA, the activated enzyme cleaves the DNA linkers on the membrane and releases the Au–Fe_3_O_4_ PMNs into solution. These released PMNs are then magnetically collected and transferred into a TMB–H_2_O_2_ mixture, where they catalyze color formation that can be quantified by a smartphone. This CRISPR‐regulated colorimetric platform detects resistance genes at levels below 0.1 CFU µL^−1^ and completes the full workflow within 1 h.

Beyond these solution‐phase colorimetric systems, a more direct strategy has also been developed to detect influenza B virus (IBV) directly on clinical nasopharyngeal swabs (Figure 10G–I) [155]. In this approach, a bimetallic MIL‐101(CoFe) MOF PMN with enhanced peroxidase‐like activity is functionalized with anti‐IBV monoclonal antibodies. When a virus‐containing swab is immersed in this antibody‐modified nanozyme solution, the viruses on the swab surface specifically capture and enrich the nanozymes through antigen–antibody binding. After enrichment, the swab is transferred into a TMB–H_2_O_2_ solution to develop color on the swab. At the same time, the oxidized TMB generates a strong Raman peak at 1609 cm^−1^, and this signal is further enhanced due to the semiconducting‐like properties of the MOF nanozyme. With this dual colorimetric and Raman readout, IBV can be visually identified at concentrations of 8.19 µg mL^−1^ for the FluB antigen protein and 1.03 mg mL^−1^ for the virus, while the Raman detection mode achieves a limit of 1.3 ng mL^−1^ at 5 min.

## 6 Artificial Intelligence in PMN Design and Diagnostics

**FIGURE 11:**
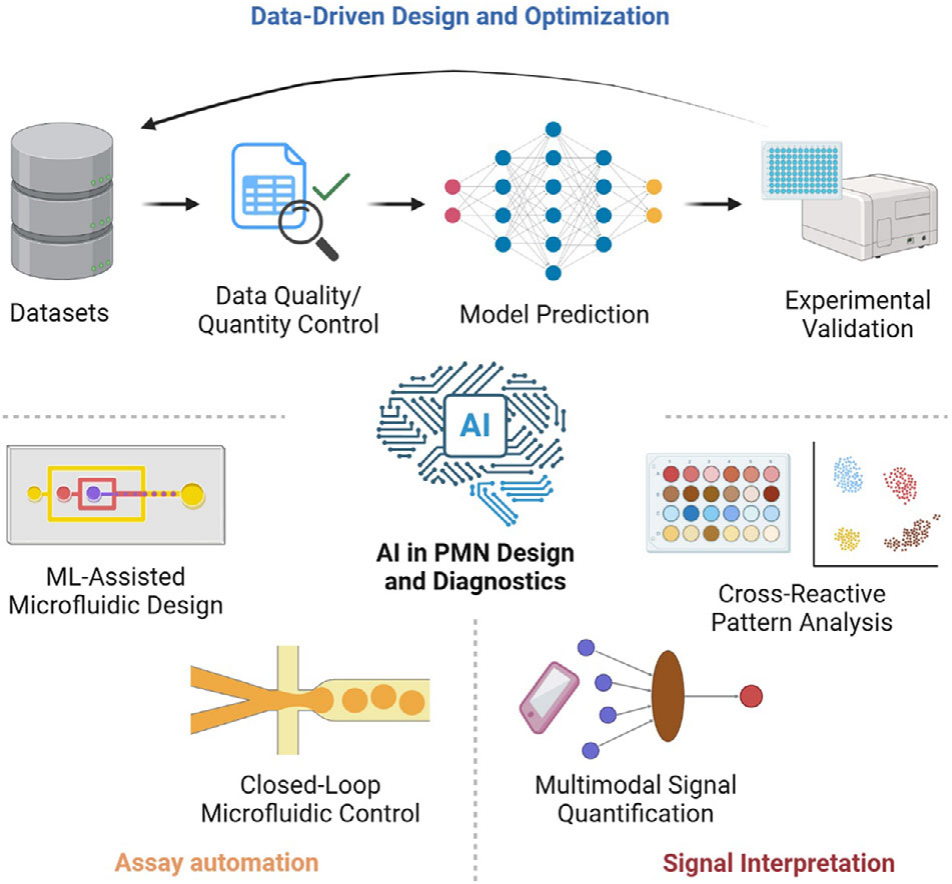
Schematic illustration of artificial intelligence (AI)‐guided PMN design and diagnostics. AI is integrated across data‐driven material design and diagnostic workflows, enabling iterative optimization of PMNs as well as automated assay operation and robust signal interpretation. Created with BioRender.com.

As the structural complexity of PMNs and functional diversity of their diagnostic platforms continue to expand, traditional trial‐and‐error approaches become increasingly insufficient, costly, and time‐consuming for navigating the high‐dimensional design space. Artificial intelligence (AI) and machine learning (ML), therefore, provide efficient tools to accelerate PMN development and platform optimization. In the context of infectious disease diagnostics, AI plays dual roles: first, in guiding the rational design and optimization of PMNs by learning structure–activity relationships from multivariate datasets; and second, in enhancing diagnostic performance by automating assay workflow to reduce user‐dependent variability and by extracting diagnostically relevant information from multimodal or cross‐reactive sensing outputs (Figure 11). This section summarizes recent advances in both directions.

### 6.1 Data‐Driven Design and Optimization

Building on the complex structure–activity relationships discussed in Section 2, traditional trial‐and‐error strategies are unlikely to keep pace with the growing design space of PMNs. AI‐assisted approaches, therefore, provide a complementary pathway for accelerating PMN design by enabling data‐driven optimization across multiple coupled variables and prioritizing promising material candidates for experimental validation [156, 157].

In a typical AI‐assisted PMN design workflow, datasets are constructed by linking nanozyme descriptors with peroxidase‐like performance metrics derived from experiments, simulations, or a combination of both [157]. Input descriptors commonly capture elemental composition, morphology, surface chemistry, defect characteristics, and reaction or assay conditions. Depending on data availability, supervised or semi‐supervised learning models are trained to establish quantitative relationships between descriptors and catalytic behavior, allowing rapid in silico screening and rational down‐selection of candidate PMN architectures. The output targets of such models typically focus on intrinsic nanozyme performance indicators, such as kinetic parameters reflecting catalytic efficiency (*V*_max_ and *K*_cat_) and substrate specificity (*K*_m_ or *K*_cat_/*K*_m_). It is noteworthy that, beyond basic descriptors, increasing attention has been directed toward the development of descriptors that capture intrinsic catalytic properties of nanozymes at a deeper level. In particular, electronic‐structure‐level descriptors including adsorption energies, reaction barriers, and orbital occupancy are frequently employed as surrogate variables that correlate with catalytic trends and can improve model predictability and transferability, although their direct physical relevance may depend on the specific material system and reaction environment. For example, *e*_g_ orbital occupancy has been identified as a predictive descriptor for peroxidase‐like activity in spinel oxide and perovskite oxide nanozymes, enabling quantitative activity prediction beyond conventional trial‐and‐error exploration [158, 159].

In view of the growing body of PMN research, increasing efforts have begun to leverage published experimental data for data‐driven analysis and prediction modeling. These studies typically construct structured databases by systematically extracting, curating, and standardizing catalytic activity data and corresponding material descriptors from existing literature. For example, the DiZyme resource was established through manual collection and curation of peroxidase‐like activity data for metal, bimetallic, metal oxide, and bimetallic oxide nanozymes. This user‐scalable database integrates comprehensive nanomaterial characterization with catalytic activity measurements for more than 300 reported nanozyme samples. Using composition‐ and physics‐informed descriptors, machine learning models trained on this curated dataset were shown to quantitatively predict peroxidase‐like activity and were further validated by successfully identifying and experimentally confirming the activities of previously unreported nanozyme compositions [160].

Simulation data derived from density functional theory (DFT) and molecular dynamics also play an important role in AI‐assisted PMN design [161, 162, 163]. In many cases, simulation results can serve as direct substitutes for experimental measurements, particularly for properties that are difficult to access experimentally or require atomic‐level precision. In data‐driven workflows, such simulation‐derived properties are often treated as reference targets during model training or large‐scale screening, with experimental measurements used for validation and refinement. In addition, the integration of simulation data provides physical constraints that help confine model predictions within meaningful regions of the design space, reducing the risk of spurious correlations.

The performance of data‐driven materials design is fundamentally constrained by both data quantity and data quality. In many materials datasets, including those related to PMNs that are primarily compiled from experimental literature, the number of available samples is often limited due to labor‐intensive synthesis, characterization, and catalytic evaluation, whereas the associated descriptor spaces can be high‐dimensional. This imbalance between sample size and feature dimensionality poses a major challenge for model robustness and generalization. Recent studies have therefore emphasized the concept of data quantity governance, in which sample quantity and feature dimensionality are jointly governed rather than optimized independently [164]. Within this framework, domain knowledge (e.g., descriptor importance, rule‐based relations, physical models, or knowledge graph representations) plays a central role in guiding descriptor selection, dimensionality control, and model parameterization to achieve balanced and physically meaningful representations.

Beyond data quantity, data quality has emerged as a critical determinant of reliable machine learning outcomes in materials research. Datasets aggregated from heterogeneous sources often contain inconsistencies arising from measurement uncertainty, varying experimental protocols, unit conversion errors, or incomplete metadata, which can introduce anomalous data points and obscure genuine structure–property relationships. To address these challenges, recent materials informatics frameworks have proposed systematic data governance strategies that define what aspects of data quality should be evaluated, when governance should be applied across the data lifecycle, and how quality issues can be identified and resolved through structured processing workflows [165]. Building on these principles, domain knowledge‐assisted data anomaly detection approaches have been developed to complement purely data‐driven methods. By incorporating physical constraints, empirical boundaries, and known correlations between descriptors, such workflows enable multilevel identification of anomalous values, followed by targeted correction or exclusion. Importantly, integrating materials domain expertise into data quality control has been shown to improve both dataset reliability and downstream model performance, indicating that robust data governance is a prerequisite for effective AI‐assisted materials design and optimization [166].

### 6.2 Automated Control and Signal Interpretation

Beyond the nanozyme design, AI is reshaping the operation of rapid diagnostic platforms themselves. In automated microfluidic systems, AI‐driven control of valve switching, flow‐rate regulation, and timing coordination has been shown to improve the precision of sample handling and allow real‐time adaptation to variations in sample viscosity, particle load, or microbubble formation [167]. At the design stage, this trend is illustrated by machine‐learning‐assisted design automation in continuous‐flow droplet microfluidics [168]. In such workflows, experimentally measured droplet diameters and generation rates are collected across a wide range of device geometries, flow‐rate ratios, and fluid properties, and used to train supervised regression models. After validation through blind prediction on previously unseen fluids and geometries, these models enable inverse specification of operating parameters. This allows target droplet dimensions and throughputs to be achieved without manual tuning, thereby reducing operator‐to‐operator variability during device setup. At the operation stage, AI is instead embedded directly into the control loop, for example, in the AI‐assisted digital microfluidics system µDropAI [169]. In this case, real‐time image streams of droplets serve as input data for image‐based state recognition models based on U‐Net semantic segmentation architectures, which are trained to classify manipulation states, including successful splitting, incomplete division, or merging. These state classifications are validated against experimentally observed manipulation outcomes and are directly coupled to feedback control rules that dynamically adjust actuation timing or voltage sequences when failures are detected. This enables self‐correcting droplet manipulation under sample‐dependent perturbations during assay execution.

AI is also transforming the analytical dimension of nanozyme‐based sensing, particularly by redefining how signals are read, processed, and interpreted. A first and immediate impact lies in improving the accuracy and robustness of signal readout. For example, an AI‐assisted immunoassay for cardiac troponin I (cTnI) protein using hollow Prussian blue PMNs as catalytic labels for TMB oxidation and as photothermal transducers in response to near‐infrared irradiation at the same time [170]. The corresponding colorimetric and photothermal signals were simultaneously acquired using a smartphone‐adapted platform. A parallel 3‐layer artificial neural network (ANN) with 64 hidden neurons was constructed to process these bimodal signals, tracking absorbance at 650 nm, initial temperature, end‐state temperature, and averaged photothermal response as input parameters. Through nonlinear normalization and regression of these coupled signals, the ANN enabled accurate quantification of cTnI over a wide dynamic range from 0.02 to 20 ng mL^−1^ with a detection limit of 10.8 pg mL^−1^. By integrating PMN‐catalyzed signal amplification with AI‐based multimodal data processing, this approach effectively reduced operator dependence and mitigated errors arising from manual signal interpretation.

Beyond improving accuracy, AI‐enabled pattern recognition enables high‐throughput and multiplex screening, which is particularly critical for infectious disease diagnostics. In large‐scale screening scenarios, the analytical challenge often lies in rapidly distinguishing multiple disease‐related biomarkers or infection states rather than detecting a single analyte with maximum sensitivity. Nanozyme sensor arrays, when combined with machine learning, address this need by generating multidimensional response patterns that can be classified in parallel. A representative example is a colorimetric sensor array constructed from four metal ion‐doped carbon dot PMNs (Fe‐, Mn‐, Cu‐, and Cr‐doped CDs), each exhibiting distinct peroxidase‐like activities and inhibition behaviors toward thiol‐containing metabolites [171]. Eight disease‐relevant biothiols differentially suppressed the TMB–H_2_O_2_ oxidation catalyzed by each nanozyme channel through competitive redox and surface coordination interactions. The resulting cross‐reactive inhibition profiles were converted into multidimensional colorimetric “fingerprints”. Linear discriminant analysis and hierarchical clustering analysis enabled clear clustering and qualitative discrimination of eight structurally similar thiols, while other supervised machine learning models further improved classification robustness and prediction accuracy. Together, these approaches enabled reliable discrimination of biothiols with a detection limit of 40 nM.

Importantly, an AI‐assisted pattern recognition strategy also provides a route to reduce reliance on target‐specific bioaffinity modification of nanozymes in bioassays, which often increases cost, introduces batch‐to‐batch variability, and may compromise catalytic activity or stability. In contrast to conventional lock‐and‐key recognition, AI‐driven PMN sensor arrays exploit cross‐reactive interactions between nanozymes and analytes, with selectivity emerging at the data‐analysis stage rather than from molecular recognition. For instance, a colorimetric sensor array based on Fe–N–C single‐atom nanozymes and machine learning was shown to differentiate multiple foodborne bacterial pathogens, including *Salmonella*, *Staphylococcus aureus*, *Vibrio vulnificus*, *Vibrio Harvey*, *Vibrio parahaemolyticus*, and *Listeria monocytogenes*, without using pathogen‐specific antibodies [172]. In this system, different pathogens differentially inhibited the peroxidase‐like activity of Fe–N–C PMNs toward three chromogenic substrates (TMB, OPD, and ABTS), generating distinct multichannel inhibition patterns. These pathogen‐specific response fingerprints were classified with high accuracy using multivariate pattern‐recognition algorithms, allowing simultaneous identification of multiple pathogens over a concentration range of approximately 10^5^–10^8^ CFU mL^−1^. This shift from molecular specificity to algorithm‐enabled selectivity highlights how AI can expand the diagnostic scope of nanozyme systems while simplifying assay design.

## 7 Cost Effectiveness and Societal Impact

Production of analytical‐grade natural peroxidases (e.g., HRP) suitable for biosensing remains costly, owing to the complexity of extraction and purification processes and the stringent facility requirements needed to ensure enzyme quality. Moreover, maintaining the enzymatic activity of peroxidases during storage and transport requires tight environmental controls (e.g., low temperature and stable humidity), leading to inconvenience and substantial added logistical costs. In contrast, PMNs, especially those composed of low‐cost metal compounds or carbon‐based materials, offer strong potential for cost‐effective large‐scale production due to their intrinsic material and manufacturing advantages. First, PMNs can be synthesized using simple and economical processes. Many of them are produced through solution‐phase methods using readily available chemicals and reagents [37]. Purification of PMNs from a synthesis can be achieved through inexpensive techniques such as filtration and centrifugation. Green synthesis approaches provide even more affordable and sustainable alternatives compared with conventional chemical methods [173]. Furthermore, recent advancements in nano‐manufacturing technologies, such as continuous‐flow production enabled by droplet‐based microreactors [174, 175], may enable scalable production while improving batch‐to‐batch consistency. Although these approaches are still emerging, they illustrate the feasibility of manufacturing PMNs through streamlined processes that contrast with the resource‐intensive production of natural peroxidases. Importantly, most PMNs exhibit excellent stability at ambient temperatures and do not require cold‐chain storage or transportation [176], significantly reducing logistical barriers and enhancing compatibility with decentralized testing environments. Their robustness enables prolonged use and supports multiple catalytic cycles, improving cost efficiency on a per‐assay basis. Considering these attributes, PMNs are positioned as a cost‐effective alternative to natural peroxidases for manufacturing diagnostic kits for infectious disease detection.

PMN‐based detection platforms hold great potential for improving the affordability and scalability of diagnostic testing. They support assay formats that do not require highly trained personnel or specialized laboratory infrastructure (see examples in Section 5 above). Their ability to integrate with simple visual outputs or smartphone‐assisted readouts further minimizes the need for technical expertise, allowing tests to be conducted in community settings or at the point of need [177]. Because PMNs are stable under ambient conditions, their use could simplify distribution systems and support wider implementation in field settings, mobile clinics, and remote communities where maintaining controlled environments remains challenging [178]. If deployed in practice, PMN‐enabled assays may aid in earlier case identification and more timely clinical decision‐making, which are critical for reducing severe disease progression and helping avoid unnecessary healthcare utilization and treatment costs associated with advanced symptoms. Collectively, these advantages could lower per‐test operational costs and help enable widespread, accessible testing capacity.

The scalability and potentially low unit cost of PMN‐based diagnostics make them promising tools for high‐frequency testing and, eventually, population‐level screening. This capability would be particularly valuable for identifying and containing transmissible diseases during epidemics or outbreaks, where delays in detection can accelerate transmission and amplify healthcare and economic burdens. With minimal resource requirements and high portability, these assays could, in principle, be distributed across diverse public spaces, such as schools, workplaces, airports, community health centers, and residential settings (e.g., nursing homes) [179, 180]. Such advantages may be especially impactful in low‐ and middle‐income countries, where conventional enzyme‐based tests can be prohibitively expensive and diagnostic capacity is often constrained by limited laboratory infrastructure [177, 181]. The stability and long shelf life of PMNs also suggest their suitability for emergency reserves or stockpiling, a useful attribute particularly in settings prone to supply chain disruptions or constrained laboratory services [179].

From a societal and public health perspective, PMN‐based diagnostics offer a promising opportunity to broaden timely access to testing and contribute to narrowing health disparities across diverse populations. By reducing reliance on centralized laboratories and lowering both direct costs and logistical burdens, PMN‐enabled platforms could help overcome persistent barriers commonly faced by underserved, rural, or remote communities, such as limited clinic or diagnostic availability, weak infrastructure, and shortages of trained personnel [182, 183, 184]. Their potential applicability in rapid response to emergency conditions (e.g., natural disasters, humanitarian crises, or periods when healthcare systems are overwhelmed) adds further value by supporting continued access to essential diagnostic services when traditional infrastructure is compromised. The ability to conduct early, on‐site testing may enable quicker identification of potential cases and inform targeted public health interventions, such as isolation guidance, resource allocation, and other rapid response measures, thereby contributing to more timely and coordinated response efforts.

The simplicity, portability, and low‐resource requirements of PMN‐enabled assays make them well‐suited for home‐ and community‐based testing programs. These decentralized approaches can empower individuals to engage in self‐monitoring and make informed decisions (e.g., whether to seek care or to isolate when necessary). Home testing can help reduce stigma and address logistical barriers such as transportation limitations, long wait times, or financial constraints—obstacles that have been shown to cause missed or delayed care in many communities [183, 185, 186]. Providing rapid results may also help alleviate anxiety and bolster individuals’ confidence in public health guidance. Importantly, accessible home testing expands diagnostic reach to individuals who might otherwise face difficulties accessing formal testing or healthcare, thereby offering meaningful potential to reduce undetected transmission and support broader disease control efforts.

Looking ahead, PMN‐based technologies hold promise for enhancing public health surveillance systems and supporting more responsive disease monitoring. Their compatibility with digital health tools (e.g., smartphone readers, automated data capture, and cloud‐based reporting) suggests potential for integration into real‐time monitoring networks. Such platforms could support early detection of emerging pathogens, more agile public health responses, and improved disease surveillance in geographically dispersed or resource‐limited regions. While substantial work remains to validate PMN‐based diagnostics in clinical settings, as well as optimizing their performance and ensuring regulatory compliance, the foundational attributes of PMNs indicate meaningful opportunities for future application and scale‐up.

Overall, PMN‐based diagnostic technologies offer a compelling combination of potential economic and societal benefits. With continued innovation, rigorous evaluation, and thoughtful integration into healthcare systems, PMN‐enabled platforms may contribute to more affordable, accessible, and resilient infectious disease detection across a broad range of global settings.

## 8 Challenges and Opportunities

This review summarizes current strategies for designing and synthesizing PMNs and discusses their use in rapid detection platforms specifically developed for infectious disease‐related biomarkers detection. These PMN‐based biosensing technologies have demonstrated distinct advantages, including high sensitivity, robustness, short assay time, and low cost, which collectively support their strong potential for POC diagnostics. Despite these advances, several scientific and practical challenges remain, continuing to affect the reliability and broader translation of these systems.

### 8.1 Batch‐to‐Batch Reproducibility

Reproducibility remains one of the most frequently reported issues because the catalytic activity of PMNs depends strongly on the structural and chemical precision of nanomaterials. During the preparation of PMNs, small variations in elemental composition, oxidation state, crystal facets, porosity, or particle size distribution of the PMNs can cause significant changes in their catalytic output. Additional variability arises during assembly of the PMN‐based biosensors, including antibody or aptamer conjugation, membrane coating, washing, drying, centrifugation, and storage. During specimen handling, solution conditions for pretreatment, such as pH, ionic strength, and temperature, can also introduce another layer of variability and thus influence sensing performance. Therefore, establishing standardized manufacturing and operating procedures is important for achieving consistent performance.

### 8.2 Catalytic Specificity

Compared with natural peroxidases, PMNs generally exhibit lower substrate selectivity. Many PMNs also possess oxidase‐like or catalase‐like activities, which can interfere with the intended catalytic reactions during detection. For instance, oxidase‐like activity may oxidize chromogenic substrates using dissolved oxygen and introduce additional background signal, whereas catalase‐like activity may decompose H_2_O_2_ under alkaline conditions or elevated temperatures, thereby diminishing the expected color development [187]. Although these side reactions may not alter the final detection result, they can bias signal intensity and compromise measurement accuracy, particularly in assays that rely on precise quantification. Improving specificity and suppressing competing pathways remain, therefore, major scientific challenges.

### 8.3 Nonspecific Interactions

The high catalytic efficiency of PMNs increases the likelihood of background color formation due to nonspecific adsorption onto proteins, lipids, or other biomolecules in real samples. These unintended reactions may partially overlap with true signals and complicate visual interpretation, which is particularly important for qualitative POC readouts. Although calibration curves can correct quantitative measurements, reducing nonspecific binding and matrix interference remains important for achieving reliable results.

### 8.4 Sample Pretreatment and Matrix Effects

Clinical specimens such as sputum, stool, and whole blood contain complex biomatrices. High protein content, mucins, salts, and cellular components can reduce the binding efficiency of bioreceptors such as antibodies, aptamers, and nucleic acids. Proteins and biomolecules can also adsorb onto PMN surfaces and form a protein corona that can shield catalytic sites or alter catalytic behavior in unpredictable ways [188]. Moreover, biosafety pretreatment steps such as filtration, lysis, or inactivation can influence assay performance. They may change the concentration or binding‐related structural features of target biomarkers, alter solution conditions by introducing detergents that affect the catalytic activity of PMNs, or release matrix components that increase background signals. Because PMN‐based sensing platforms operate through surface catalysis and differ fundamentally from conventional enzyme assays, these pretreatment methods require further practical refinement to better accommodate this emerging technology.

### 8.5 Post‐Use Disposal and Biosafety

Many PMNs contain metal‐based components or poorly degradable structures that may exert cytotoxic or environmental effects after disposal. In addition, diagnostic devices used for infectious disease detection can retain infectious contents that pose secondary exposure risks. Therefore, safe waste management, appropriate inactivation procedures, and environmentally considerate PMN design will be important considerations for large‐scale deployment.

Though PMN‐based rapid sensing platforms are not impeccable systems, they remain at an early stage of development and continue to evolve rapidly to accommodate increasing diagnostic demands, especially in light of the rising frequency of infectious disease outbreaks. As such, new opportunities continue to emerge that may further expand the scope, performance, and practicality of PMN‐enabled rapid diagnostics. One important direction lies in exploring signal‐generation strategies beyond the classical peroxidase‐like activity that is based on H_2_O_2_‐mediated oxidation of chromogenic substrates. For instance, oxidase‐like nanozymes that use dissolved oxygen as the electron acceptor can generate colorimetric signals without requiring unstable H_2_O_2_ [189]. In addition, several infection‐associated metabolites can be quantified through nanozyme‐mediated redox reactions. For example, glutathione, a key indicator of oxidative status that fluctuates during infection and inflammation, can readily reduce the blue oxidized form of TMB to its colorless state, enabling reverse colorimetric detection when coupled with nanozyme catalysis [190].

Another important direction in PMN‐enabled rapid diagnostics is the development of cascade reaction systems that enable the detection of biomarkers inaccessible to single‐step PMN assays. By combining PMNs with upstream oxidases or auxiliary reactions, cascade amplification expands both the analyte scope and sensitivity of rapid diagnostic platforms. However, for quantitative detection, and especially in cascade colorimetric assays, preserving nanozyme reaction specificity becomes critical. Because different enzymatic steps often operate optimally under distinct pH conditions and many PMNs simultaneously exhibit oxidase‐ or catalase‐like activities, unintended side reactions can bias signal intensity and compromise measurement accuracy. Breaking pH‐related selectivity constraints has therefore emerged as a central challenge in PMN‐based cascade diagnostics. A range of strategies has been explored to address this challenge. One representative approach relies on spatial separation of incompatible reaction conditions while still allowing the catalytic steps to proceed in sequence [191]. A clear illustration of this concept is provided by a foldable paper‐based microfluidic device, in which glucose oxidation and peroxidase‐like signal generation are carried out in physically distinct regions of the same paper strip under different local pH environments. In this design, the sample is first introduced into an upstream zone where glucose oxidase operates at near‐neutral pH to produce H_2_O_2_. Upon folding the paper, the generated H_2_O_2_ is brought into contact with a downstream compartment preconditioned to an acidic pH and containing a single‐atom iron‐site‐containing hydrogel PMN, where selective TMB oxidation occurs. By using the folding step to connect two otherwise incompatible reaction environments, the system effectively preserves optimal conditions for each catalytic step without additional instrumentation or buffer exchange. A second strategy focuses on surface modification to improve reaction selectivity in cascade assays operating under near‐neutral conditions. In a representative system, heparin‐functionalized Pt nanoclusters were used in conjunction with glucose oxidase for colorimetric glucose detection [192]. The surface‐bound heparin, a highly negatively charged polysaccharide, enhances the local accumulation of the positively charged TMB substrate near the Pt surface through electrostatic interactions, thereby favoring the peroxidase‐like reaction pathway. As a result, the Pt PMNs effectively minimize oxidase‐like interference and enable quantitative cascade detection with minimal dissolved‐oxygen background. Another emerging strategy involves direct regulation of the coordination environment at the active‐site level. In a representative example, Ru‐centered single‐atom PMNs were engineered to retain out‐of‐plane chlorine ligands at the Ru sites (RuNC_Cl) [193]. DFT calculations combined with Bader charge analysis reveal that this coordination geometry induces repulsive interactions against secondary H_2_O_2_ adsorption owing to the valence orbital occupancy of the Ru center and the presence of two axial Cl ligands, thereby increasing the energy barrier for the catalase‐like reaction. As a result, the RuNC_Cl PMN exhibits strongly peroxidase‐selective behavior under near‐neutral conditions, with peroxidase‐like activity approximately 38‐fold higher than catalase‐like activity, enabling one‐pot cascade assays for quantitative biomarker detection without buffer exchange.

In addition to improving reaction specificity, an important opportunity for advancing PMN‐based rapid diagnostics lies in enhancing the controllability and reproducibility of the overall detection workflow. Automation and data‐driven analysis offer powerful routes to reduce user‐dependent variability, particularly in integrated microfluidic systems where assay timing, signal acquisition, and decision thresholds strongly influence quantitative outcomes. At the same time, AI‐assisted material design and assay automation rely on large, well‐curated datasets, whose scale and diversity are difficult to achieve within a single laboratory [194]. This dependence creates a natural pressure toward greater consistency in data generation, evaluation, and reporting, thereby motivating the development of standardized benchmarking frameworks and harmonized experimental protocols. While PMN synthesis, catalytic activity evaluation, and bioassay workflows are currently implemented using diverse and often laboratory‐specific practices, the increasing use of AI‐driven methods favors data that are comparable and interoperable across studies. As a result, the demand for reliable AI training is expected to accelerate convergence toward shared benchmarking practices, facilitating cross‐laboratory comparability and supporting the robust translation of PMN‐based diagnostic platforms into practical, scalable applications.

In conclusion, PMNs represent a powerful and rapidly evolving class of catalytic materials for infectious disease detection. Their high catalytic efficiency, robustness, and distinctive physicochemical properties position them as promising alternatives to conventional enzymatic reporters in rapid sensing platforms, while their emerging integration with automation and data‐centric analysis opens new opportunities for next‐generation diagnostics. We hope this review offers a consolidated overview of recent advances and helps inform future innovations in nanozyme design, sensing strategies, and translational applications.

## Conflicts of Interest

The authors declare no conflicts of interest.

## Data Availability

The authors have nothing to report.
